# Intravenous-to-enteral drug substitution in the ICU: a single-centre analysis of the potential to reduce the environmental footprint of critical care

**DOI:** 10.1186/s13054-026-06331-z

**Published:** 2026-09-22

**Authors:** Clémence Marois, Maxime Gasperment, Adam Celier, Ambre Preteux, Grégoire Gaucher, Léontine Scherer, Nicolas Weiss, Kevin Bihan, Marie Lecronier, Amélie Liou

**Affiliations:** 1 AP-HP, Sorbonne Université, Hôpital de la Pitié-Salpêtrière, Service de médecine intensive et réanimation neurologique, 47-83, Bd de l’hôpital, Paris, 75013 France; 2 AP-HP, Sorbonne Université, Hôpital de la Pitié-Salpêtrière, Service d’Hépato-Gastro-Entérologie, Paris, France; 3 AP-HP, Sorbonne Université, Direction des Achats du Développement Durable et de la Logistique, Paris, France; 4 Faculté d’Économie, Université de Montpellier, Montpellier, France; 5 AP-HP, Sorbonne Université, Hôpital de la Pitié-Salpêtrière, Service de Pharmacie, Paris, France; 6 Department of Pharmacology, Regional Pharmacovigilance Center, Groupe Hospitalier Pitié Salpêtrière, Assistance Publique-Hôpitaux de Paris, INSERM, Sorbonne University, CIC-2503, Paris, France; 7 AP-HP, Sorbonne Université, Hôpital de la Pitié-Salpêtrière, Service de médecine intensive réanimation, Paris, France

**Keywords:** IV-to-enteral switching, Sustainability, Carbon footprint, Intensive care

## Research letter

Intravenous (IV) medications account for most medication-related greenhouse gas emissions in the intensive care unit (ICU) because they require infusion bags, tubing, syringes, carrier fluids and other single-use devices [1, 2]. Unnecessary IV therapy also carries an avoidable burden of administration errors, adverse drug events, and fluid and sodium load [3–6]. Yet IV therapy is often continued despite many patients having conditions compatible with enteral drug administration. We therefore aimed to describe, in a single centre, which IV medications have a feasible enteral alternative, to propose a pragmatic classification based on pharmacokinetic, safety and efficacy data, and to estimate the potential environmental and economic impact of such a strategy.

All IV medications dispensed during 2024 across eight medical and surgical ICUs (186 beds) of a French university hospital were reviewed. For each drug we assessed the availability of an enteral formulation and the feasibility of administration through feeding tubes. Patient-level clinical eligibility for an enteral switch was considered the responsibility of the treating clinician, who should assess if the drug is still indicated, whether enteral administration is clinically appropriate and reliable, taking into account organ dysfunction and haemodynamic stability, adequate gastrointestinal tract function, the absence of major factors likely to impair enteral absorption. A multidisciplinary panel (four intensivists, three clinical pharmacists, one nurse) then classified medications against criteria covering expected absorption, drug-specific properties, tolerance and efficacy data for the oral formulation when available. Two categories were used: (1) Ready for IV-to-enteral substitution, switchable once enteral administration is judged reliable after individual clinical assessment; (2) Requires monitoring before substitution, feasible but requiring therapeutic drug monitoring or close clinical assessment because of variable pharmacokinetics. Environmental impact and medication acquisition costs were modelled for the five most frequently dispensed eligible medications using process-based life-cycle assessment. Additional methodological details are provided in the Supplementary Appendix.

**Fig. 1:**
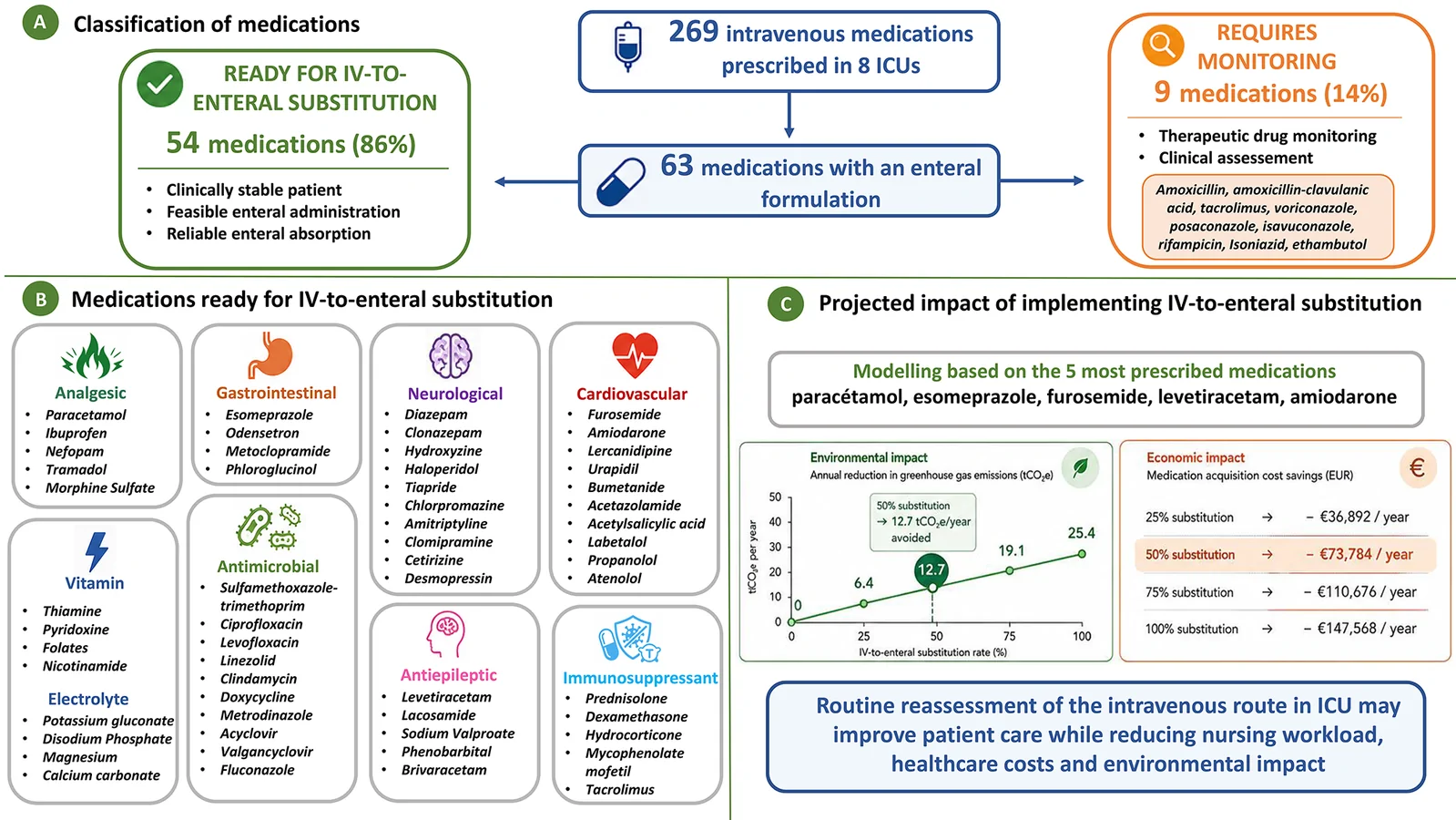
Classification of intravenous medications according to their suitability for IV-to-enteral substitution and projected environmental and economic impact of implementation. Of 269 intravenous medications prescribed across participating ICUs in 2024, 63 had a feasible enteral alternative. Based on pharmacokinetic characteristics, safety, efficacy, available recommendations, medications were classified as either Ready for IV-to-enteral substitution (54 medications) or Requires monitoring before substitution (9 medications). Panel B lists the 54 medications classified as Ready for IV-to-enteral substitution; substitution always requires individual clinical assessment and confirmation that the drug remains indicated. Environmental and economic modelling was performed for the five most frequently dispensed medications classified as Ready for IV-to-enteral substitution (paracetamol, a proton pump inhibitor [intravenous pantoprazole substituted by enteral lansoprazole], furosemide, levetiracetam and amiodarone). The graph shows the estimated annual reduction in greenhouse gas emissions according to increasing rates of IV-to-enteral substitution (25%, 50%, 75% and 100%); corresponding medication cost savings are summarised alongside the graph. At 50% substitution these projections correspond to 0.19 kgCo₂e and €1.09 per ICU bed-day (67,890 bed-days, assuming full occupancy of 186 beds over one year). All figures are modelled projections derived from pharmacy dispensing data and do not represent measured savings

Of 269 IV medications dispensed, 63 had a feasible enteral alternative (Table S1). Fifty-four (86%) were classified as Ready for IV-to-enteral substitution and nine (14%) as Requires monitoring before substitution because of unpredictable absorption or the need for therapeutic drug monitoring (Table S2). Medications Ready for IV-to-enteral substitution included analgesics, gastrointestinal and psychotropic drugs, antiepileptics, cardiovascular agents, corticosteroids and immunosuppressants, antimicrobials, vitamins and electrolytes (Fig. 1); those Requiring monitoring were mainly antimicrobials and immunosuppressants. The five most frequently dispensed eligible medications (paracetamol, a proton pump inhibitor, furosemide, levetiracetam and amiodarone) together generated 37.7 tCO₂e and €152,206 in acquisition costs annually. Assuming that 50% of IV administrations were replaced by enteral formulations, annual greenhouse gas emissions would decrease by 12.7 tCO₂e and medication costs by €73,784, corresponding to 0.19 kgCO₂e and €1.09 per ICU bed-day (67,890 bed-days at full occupancy). Complete substitution would double these projected reductions (Fig. 1).

This single-centre analysis of dispensing data describes the scale of the opportunity for IV-to-enteral substitution at system level; it does not establish whether a switch should occur in an individual patient. Beyond its environmental implications, systematic reassessment of the IV route is primarily a patient-centred quality-improvement strategy: it shortens exposure to vascular access devices, avoids unnecessary fluid and sodium load, reduces the errors and adverse events inherent to parenteral administration, and lessens nursing workload [3–6]. As with antimicrobial stewardship, the daily medication review should first establish whether the drug remains indicated and can be stopped, and only then whether the route can be de-escalated [7]. Enteral absorption is nonetheless variable in critical illness because of altered splanchnic perfusion, impaired gastrointestinal motility, changes in gastric pH, and drug-drug and drug-feed interactions, the latter sometimes requiring feeding interruptions with consequences for glycaemic control and nutritional target attainment [8, 9]. Crushing tablets or opening capsules is frequently outside the marketing authorisation and must follow validated pharmaceutical references [10]. The proposed classification is intended to support, not replace, bedside judgement, and should be regarded as hypothesis-generating pending prospective validation of an IV-to-enteral switch tool at national or international level.

This retrospective, single-centre analysis used pharmacy dispensing data, which overstate administration because dispensed units may be wasted, returned or transferred. No patient-level safety, therapeutic drug monitoring or clinical outcome data were available to confirm therapeutic equivalence after switching, and the safety data underpinning the classification derive largely from non-ICU populations. The most frequently dispensed medications are not necessarily those with the greatest environmental or economic impact, so the modelled figures should not be read as the maximum achievable benefit. Drug availability varies between countries, environmental estimates relied on modelling, and achievable substitution rates depend on patient eligibility and local implementation. Baseline switch rates depend on daily clinical pharmacist review, the provision of which varies widely between countries [11], limiting the transferability of the projected gains. Strengths include the exhaustive inclusion of all IV medications dispensed across eight ICUs over a full year, multidisciplinary consensus classification, and life-cycle assessment applied to real consumption data.

IV-to-enteral stewardship is a simple candidate quality-improvement intervention combining patient safety, cost reduction and environmental sustainability. Development of local protocols and prospective validation of a switch tool should now be priorities for sustainable critical care.

## Take-home message

Nearly one quarter of intravenous medications dispensed in our ICUs had a feasible enteral alternative. Systematic reassessment of the route of administration may reduce adverse drug events, unnecessary fluid and sodium load, plastic waste, nursing workload and the environmental footprint of critical care, and warrants prospective evaluation.

## Electronic supplementary material

Below is the link to the electronic supplementary material.

Supplementary Material 1

Supplementary Material 2

Supplementary Material 3

Supplementary Material 4

Supplementary Material 5

Supplementary Material 6

## Data Availability

No datasets were generated or analysed during the current study.

## Abbreviations

GHG: Green House Gas
AP-HP: Assistance Publique - Hôpitaux de Paris
CO₂: Carbon dioxide
ICU: Intensive care unit
IV: Intravenous
kgCO₂eq: Kilograms of carbon dioxide equivalents
tCO₂eq: Tons of carbon dioxide equivalents
LCA: Life cycle assessment
