# Supplementary material for: Intravenous-to-enteral drug substitution in the ICU: a single-centre analysis of the potential to reduce the environmental footprint of critical care
Source: Crit Care. 2026 Sep 22;30:487. doi: 10.1186/s13054-026-06331-z (PMC13599322; doi:10.1186/s13054-026-06331-z)
Supplement: Supplementary file 1 [file 13054_2026_6331_MOESM6_ESM.pdf]

# CAREBONE

## Outil d'estimation de l'empreinte carbone d'un parcours patient

Guide méthodologique

PROJET

**Version Médicaments & Dispositifs médicaux uniquement**

| | |
|---|---|
| **Département** | Transformation Ecologique et Santé Environnementale |
| **Date dernière mise à jour** | 25/01/2024 |

## Contexte et objectif

Les émissions de gaz à effet de serre du secteur de la santé ont été estimées à environ 8%[1] des émissions françaises, avec 49 millions de tonnes de $CO_2e$ par an.

Un grand nombre d'établissements de santé s'engagent sur le volet climatique et réalisent leur **Bilan Carbone®** ; ce qui donne une perspective globale de leurs émissions de GES. L'AP-HP a voulu aller encore plus loin et avoir une **vue plus opérationnelle** de ses émissions tout au long d'un **parcours de soins**. La méthodologie décrite dans ce document reprend l'ensemble des formules, hypothèses et sources utilisées pour calculer l'empreinte carbone des **différents éléments** d'un parcours de soins : médicaments, dispositifs médicaux (DM) et actes médicaux.

PROJET

[1] Selon le rapport final « Décarboner la santé pour soigner durablement » du think thank français The Shift Project – édition 2023

# Table des matières


Contexte et objectif ........ 2
Fonctionnement de l'outil ........ 3
Périmètre ........ 4
Calcul de l'empreinte carbone d'un médicament ........ 6
  Méthodologie globale ........ 6
  Matériel et informations nécessaires ........ 6
  Méthodologie détaillée ........ 7
  Hypothèses & sources ........ 10
Calcul de l'empreinte carbone d'un dispositif médical ........ 18
  Méthodologie globale ........ 18
  Matériel & informations nécessaires ........ 18
  Méthodologie détaillée ........ 19
  Hypothèses & sources ........ 22
Incertitude ........ 29
Facteurs d'émissions & sources ........ 30
Protocoles transversaux utilisés ........ 39
  Nomenclature ........ 39
  Archivage ........ 40
Acronymes ........ 40
Bibliographie ........ 41


PROJET

# Fonctionnement de l'outil

L'outil est composé de différents onglets :

<u>1 onglet mauve</u> :

- "Lisez-moi" : explications méthodologiques de l'outil pour guider l'utilisateur.

<u>4 onglets bleu clair</u> :

Dans ces onglets, les cellules blanches peuvent être complétées par l'utilisateur. Les cellules mauves sont automatisées.

- "Parcours patient" : calculateur permettant d'estimer l'empreinte carbone d'un parcours patient

- "Actes" : calculateur permettant d'estimer l'empreinte carbone d'un acte (peu importe sa nature) et d'alimenter l'onglet "Parcours patient". Une fois un résultat obtenu, copier la dernière ligne "Extraction pour base de données" et la coller en valeur dans l'onglet "FE Actes"

- "Médicaments" : calculateur permettant d'estimer l'empreinte carbone d'un médicament et d'alimenter les onglets "Actes" et "Parcours patient". Une fois un résultat obtenu, copier la dernière ligne "Extraction pour base de données" et la coller en valeur dans l'onglet "FE Médicaments"

- "Dispositifs médicaux" : calculateur permettant d'estimer l'empreinte carbone d'un dispositif médical ou un consommable et d'alimenter les onglets "Actes" et "Parcours patient". Une fois un résultat obtenu, copier la dernière ligne "Extraction pour base de données" et la coller en valeur dans l'onglet "FE DM".

**Cette première version détaille uniquement la méthodologie des onglets « Médicaments » et « Dispositifs médicaux ».**

4 onglets verts – AIDE UTILISATEUR :

Ces onglets permettent d'aider l'utilisateur à estimer des données d'activité quand celles-ci ne sont pas accessibles ou à choisir une approximation de facteurs d'émission (FE) quand le FE exact n'existe pas dans la base :

- **AIDE – Choix FE API** : cet onglet permet à l'utilisateur de choisir le facteur d'émission le plus pertinent en notant la substance active (API) sur 4 critères : la complexité de la molécule, son origine, les conditions particulières de production et les volumes de production. Ces critères sont évalués de 1 (consommations énergétiques et émission GES les plus faibles) à 3 (consommations énergétiques et émission GES les plus fortes) par l'utilisateur. Celui-ci est guidé par des indications dans l'onglet mais doit aussi effectuer des recherches documentaires. Le méthodologie complète est détaillée dans Facteurs d'émissions & sources > Substances actives > Méthodologie en cas de FE inexistant.
- **AIDE – Estim qtté Excipient** : cet onglet permet à l'utilisateur d'estimer la quantité d'excipient du médicament quand celle-ci est inconnue. La méthodologie complète est détaillée dans Calcul de l'empreinte carbone d'un médicament > Hypothèses & sources > Excipients.
- **AIDE – Choix FE Excipient** : cet onglet guide l'utilisateur dans le choix du facteur d'émissions le plus pertinent. Le méthodologie complète est détaillée dans Facteurs d'émissions & sources > Excipients > Méthodologie en cas de FE inexistant.
- **AIDE – Masse matériaux DM** : cet onglet permet à l'utilisateur d'estimer la masse de matériaux des dispositifs médicaux en fonction de leurs formes et de leurs dimensions. La méthodologie complète est détaillée dans Calcul de l'empreinte carbone d'un dispositif médical > Hypothèses & sources > Matériaux.

28 onglets bleu foncé :

- 2 onglets de ratios permettant d'estimer les consommations d'énergie liées à la production de médicaments et de DM et les émissions relatives au stockage de données numériques ;
- 26 onglets correspondant à des facteurs d'émissions.

## Périmètre

Le périmètre des opérations prises en compte dans le parcours patient est décrit dans le schéma ci-dessous.

PROJET

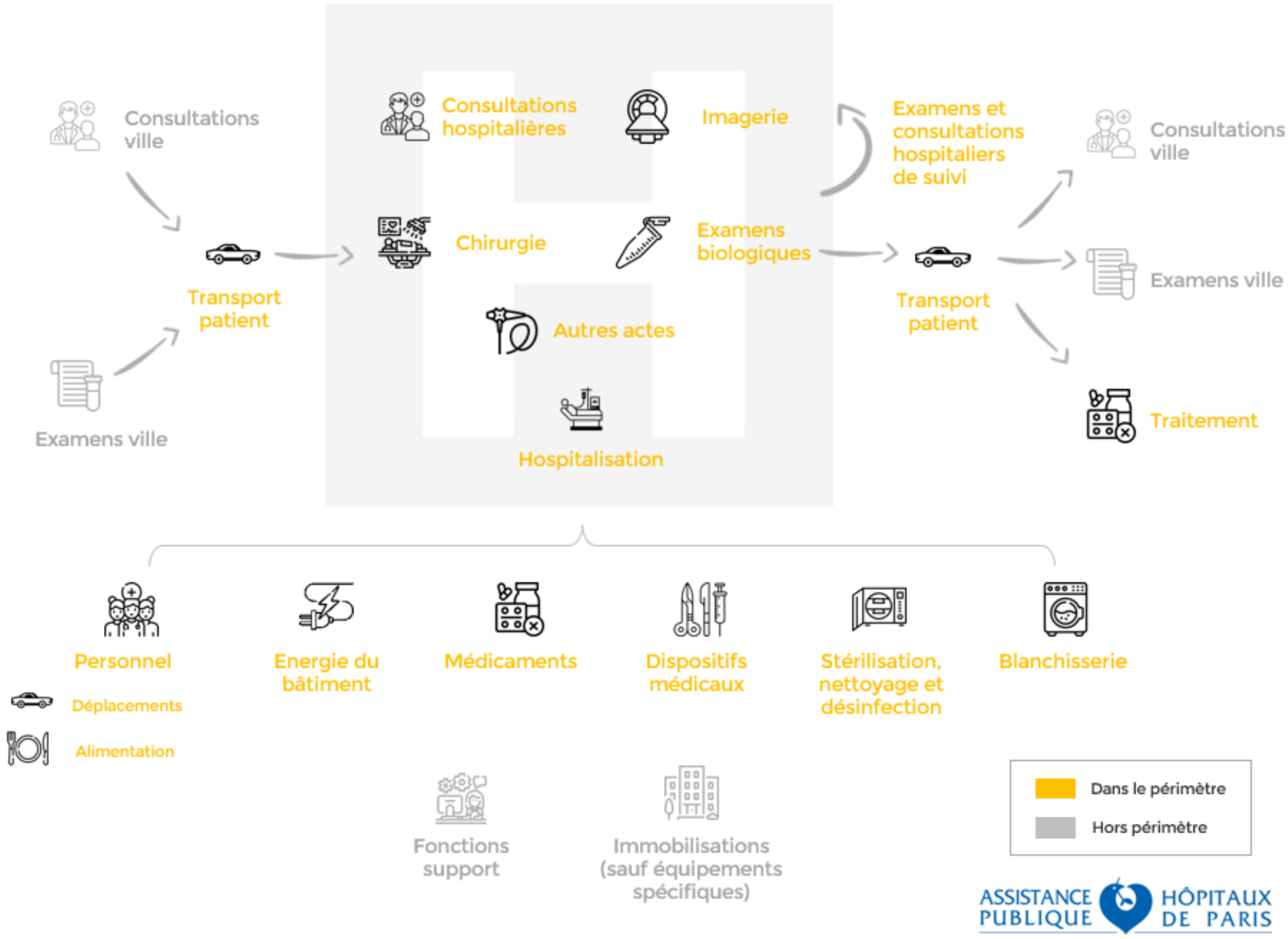


<u>Exclusions de périmètre</u>

Concernant les dispositifs médicaux et les médicaments, le périmètre se cantonne strictement au cycle de vie et ne prend pas en compte les fonctions support, la recherche & le développement, la promotion, etc.

Concernant la fin de vie des médicaments : dans un parcours de soins, le médicament est considéré comme consommé.

PROJET

# Calcul de l'empreinte carbone d'un médicament

## Méthodologie globale

Définition : Le code de la Santé publique (article L.5111-1) définit ainsi le médicament : « *toute substance ou composition présentée comme possédant des propriétés curatives ou préventives à l'égard des maladies humaines ou animales, ainsi que toute substance ou composition pouvant être utilisée chez l'homme ou chez l'animal ou pouvant leur être administrée, en vue d'établir un diagnostic médical ou de restaurer, corriger ou modifier leurs fonctions physiologiques en exerçant une action pharmacologique, immunologique ou métabolique.* » [1]

La méthodologie de calcul de l'empreinte carbone d'un médicament s'appuie sur les recommandations de la norme ISO 14040 et la méthode Bilan GES de l'ADEME. Elle est qualifiée d'approche « Analyse de Cycle de Vie (ACV) simplifiée » car un cycle de vie standard et simplifié a été modélisé (voir schéma ci-dessous). Le périmètre de cette ACV simplifiée est « du berceau à la tombe » (« cradle to grave »). Au sein de ce cycle de vie simplifié modélisé, l'utilisateur de l'outil doit paramétrer chaque étape avec les informations et données d'activité propres à un médicament donné.

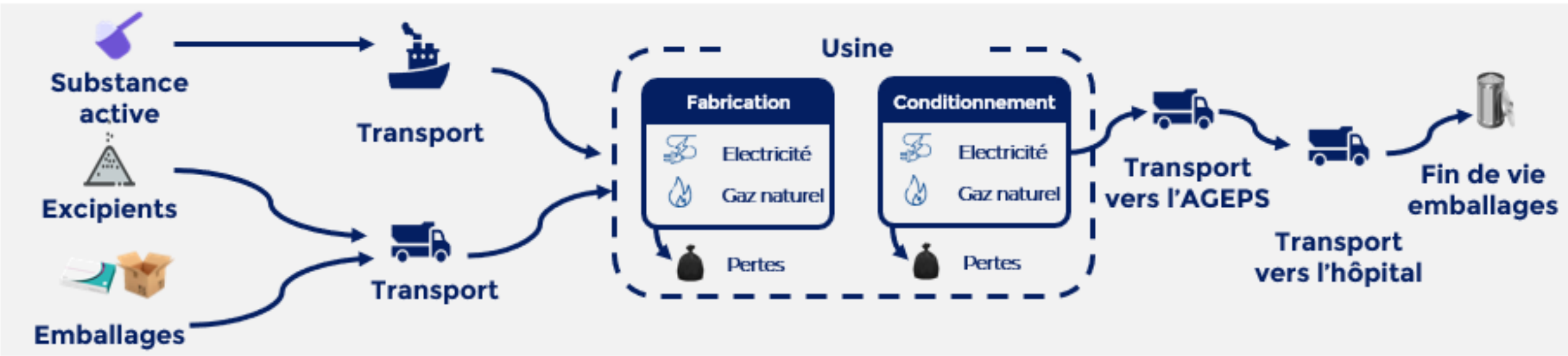


**Schéma du cycle de vie simplifié de la production et de l'utilisation d'un médicament à l'AP-HP**

La méthodologie ACV nécessite de définir l'Unité Fonctionnelle avant de procéder aux calculs. La norme ISO 14044, définit l'Unité Fonctionnelle comme suit : "*Performance quantifiée d'un système de produits, destinée à être utilisée comme unité de référence dans une ACV*". Le rôle de l'Unité Fonctionnelle est de fournir une référence par rapport à laquelle les données d'activités seront définies et normalisées afin d'assurer la comparabilité des résultats de l'ACV sur une base commune.

Dans le cadre de cette méthodologie de calcul de l'empreinte carbone d'un médicament, l'**Unité Fonctionnelle** est définie par défaut comme **la plus petite unité de médicament possible** (ex : 1 comprimé, 1 gélule, 1 ampoule, 1 flacon, etc.). La méthodologie prévoit **de nombreuses formes galéniques** possibles (comprimé, flacon, ampoule, gélule, etc.).

Les résultats des empreintes carbone de médicaments ainsi calculées alimentent une base de données de « facteurs d'émissions », accessibles à tous les utilisateurs et pour tous les parcours de soins. Ces résultats sont accompagnés d'une incertitude également enregistrée dans la base (voir « Incertitude »).

## Matériel et informations nécessaires

La réalisation de l'empreinte carbone d'un médicament nécessite de disposer :

- Du Résumé des Caractéristiques du Produit (RCP) ;
- Des informations logistiques sur le conditionnement du produit (nombre de médicaments par boîte, nombre de boîtes par carton, nombre de cartons par palette) ;
  - ⇨ Au sein de l'AP-HP, ces données peuvent être retrouvées sur le site de l'AGEPS : Marchés Equipements et Produits de santé (ap-hop-paris.fr)

- Optionnel : d'un échantillon de médicament, afin de pouvoir réaliser des pesées.

## Méthodologie détaillée

La méthodologie proposée repose sur un certain nombre de données d'activité, parfois difficiles à collecter. Si les données réelles sont disponibles / connues, il est conseillé de les saisir dans l'outil en remplacement des valeurs par défaut.

Sinon, des estimations sont proposées dans l'outil en première approche. Celles-ci sont présentées dans la partie « Hypothèses & sources ». Ces hypothèses sont fondées sur des sources explicitées également dans la partie « Hypothèses & sources ».

Pour chacun des postes ci-dessous, l'utilisateur doit saisir une donnée d'activité mais aussi sélectionner la méthodologie de collecte, c'est-à-dire la façon dont il a obtenu cette donnée. Cela permettra notamment de calculer l'incertitude des empreintes carbone ainsi estimées (voir « Incertitude »).

### Données générales

Cette catégorie permet de renseigner les informations générales du médicament dont nous souhaitons calculer l'empreinte carbone :

- Nom du médicament ;
- Format d'unité (forme galénique : gélule, sachet, comprimé, ampoule, seringue, poche, flacon, autre format) ;
- Nombres d'unités dans une boîte ;
- Conditionnement 1 – Nombre de boîtes par carton ;
- Conditionnement 2 – Nombre de cartons par palette ;
- Forme : forme liquide, sèche ou solution ;
- Numéro SAP (donnée interne à l'AP-HP) ;
- CIP 13 ;
- Nom du fabricant ;
- Responsable du calcul : Prénom et Nom de la personne en charge du calcul de cette empreinte carbone ;
- Fonction responsable : Fonction et organisation de la personne en charge du calcul ;
- Date du calcul.

### Substance(s) active(s)

Définition : Il s'agit de la substance présente dans le médicament qui lui confère ses propriétés thérapeutiques ou préventives. [2]

Les émissions liées à la fabrication des substances actives du médicament sont à calculer en relevant les quantités mentionnées sur le RCP. Ces quantités sont saisies en grammes.

### Excipients

Définition : L'excipient est un composant du médicament qui ne lui confère pas ses propriétés thérapeutiques ou préventives, mais qui peut jouer un rôle notamment dans l'absorption (assimilation) et la stabilité du médicament et conditionnant son aspect, sa couleur et son goût. [3]

PROJET

Deux méthodologies peuvent être utilisées pour les excipients en fonction des informations disponibles :

- Sur le RCP, les quantités des excipients sont parfois précisées et peuvent alors être renseignées dans l'outil.
- Sinon, l'outil propose une méthodologie d'estimation des quantités d'excipient, détaillée dans la partie « Hypothèses & sources > Excipients ».

Les quantités d'excipients sont saisies en grammes. Pour les formes liquides, la quantité d'eau pour préparations injectables (Eau PPI) est aussi renseignée en ml.

### Emballages (matériaux et transformation)

Les impacts liés à la fabrication des emballages et de la notice du médicament sont quantifiés en pesant ces éléments (grammes) ou en utilisant la méthodologie par défaut décrite dans la partie « Hypothèses & sources > Emballages ».

La masse des emballages doit être décomposée et renseignée en fonction des différents types de matériaux d'emballage (carton, papier, aluminium, plastique, …).

Le périmètre des emballages pris en compte comprend l'ensemble du conditionnement : de la palette jusqu'à la boîte de médicament. L'utilisateur précise dans l'outil si l'emballage concerne la palette, le carton, la boîte ou l'unité. Les formules de calcul permettent alors de ramener la part des émissions relatives à une unité de médicament.

Enfin, l'utilisateur choisit les facteurs d'émission correspondant aux matériaux des emballages. Il opte aussi dans le tableau suivant pour les facteurs d'émission relatifs à la transformation de ces emballages. Deux process de transformation maximum peuvent être sélectionnés par matériau.

### Transport amont de l'usine

#### *Transport des substances actives*

Ce transport correspond à la distance entre le site de production de la substance active et le site de production du médicament, exprimée en km. Les émissions de GES associées sont calculées dans l'outil en fonction de cette distance et du poids total des substances actives utilisées.

#### *Transport des excipients*

Ce transport correspond au trajet des excipients entre leur site de production et le site de production du médicament, exprimée en km. Les émissions de GES associées sont calculées dans l'outil en fonction de cette distance et du poids total des excipients utilisés.

#### *Transport des emballages*

Ces transports correspondent :

- D'une part au trajet des matériaux d'emballage jusqu'au site de production des emballages ;
- D'autre part au trajet des emballages jusqu'au site de conditionnement du médicament.

Ces distances sont exprimées en km. Les émissions de GES associées sont calculées dans l'outil en fonction de ces distances et du poids des matériaux d'emballage utilisés.

Lorsque ces informations ne sont pas accessibles, des estimations sont proposées dans la partie « Hypothèses & sources > Transport amont »

### Transport aval de l'usine

PROJET

L'outil découpe en trois étapes le transport aval de l'usine :

- Transport du médicament jusqu'à son site de conditionnement. Si celui-ci est le même que l'usine de production, la distance peut être de 0 km.
- Transport du médicament du site de conditionnement jusqu'à la centrale d'achat (l'AGEPS la plupart du temps pour l'AP-HP).
- Transport du médicament depuis la centrale d'achat jusqu'à l'hôpital.

Ces distances sont exprimées en km. Les émissions de GES associées sont calculées dans l'outil en fonction de cette distance et du poids total du médicament (substances actives, excipients et emballages).

Pour estimer les distances de transport, nous recommandons les outils suivants :

- Pour les trajets en bateaux : Online Freight Shipping & Transit Time Calculator at Searates.com
- Pour les transports routiers : Google Maps

Lorsque ces informations ne sont pas accessibles, des estimations sont proposées dans la partie « Hypothèses & sources > Transport aval»

## Formulation et conditionnement

Ce poste d'émissions correspond aux consommations énergétiques des usines de fabrication et de conditionnement du médicament. Le format de données prévu dans l'outil est un ratio énergétique, exprimé en kWh (kWh PCI pour le gaz naturel) / kg de substance active (API).

En cas de donnée indisponible, des valeurs par défaut sont proposées (voir partie « Hypothèses & sources > Formulation et conditionnement »).

## Pertes

### *Pertes de matières durant la formulation*

Lors de la production du médicament, une partie des substances actives et des excipients passe en perte ou rebus. Une part de pertes (en %) est calculée automatiquement à partir des émissions relatives à la substance active et aux excipients et est ajoutée au total des émissions.

### *Pertes de matières des emballages*

Lors du processus d'emballage, une partie des matériaux (carton, papier, aluminium, plastique, …) passe en perte ou rebus. Une part de pertes (en %) est calculée automatiquement à partir des émissions relatives aux emballages et notice et est ajoutée au total des émissions.

Des hypothèses de pourcentages de pertes sont disponibles dans « Hypothèses & sources > Pertes ».

## Fin de vie du médicament

Dans cette méthodologie, le médicament est considéré comme consommé par le patient. Ce poste a donc été exclu.

## Fin de vie des emballages et notice

PROJET

Les impacts liés à la fin de vie des emballages du médicament sont quantifiés en choisissant le mode de traitement de ces déchets : recyclage, stockage, incinération, etc. Par défaut, le facteur d'émission « Emballages - Fin de vie moyenne filière » est sélectionné en absence d'information supplémentaire.

### Empreinte carbone totale

L'empreinte carbone totale du médicament correspond à la somme des émissions de GES calculées dans chacun des postes listés ci-dessus. Un « facteur d'émission » est alors calculé est pour ce médicament dans la forme galénique et le dosage choisis. Il peut être enregistré dans la base de données afin d'être utilisé dans le calcul de l'empreinte carbone d'un acte ou d'un parcours patient (voir « Protocole d'archivage »).

### Emissions évitées dues à la fin de vie

Les émissions évitées sont calculées de manière séparée de l'empreinte carbone et sont issues de la valorisation matière et énergie des déchets. Elles correspondent aux émissions GES qui ont été évitées grâce à la non production de matière vierge et/ou à la non production d'énergie conventionnelle. Ces émissions évitées n'impactent pas le « facteur d'émission » du médicament.

## Hypothèses & sources

Un certain nombre d'hypothèses sont proposées dans cette méthodologie (distance de transport, ratios de consommations énergétiques, empreinte des emballages…). Il est conseillé d'ajuster ces estimations dans l'outil avec les données réelles du cas étudié quand celles-ci sont connues.

Les hypothèses adoptées dans cette méthodologie et leurs sources sont détaillées dans les paragraphes ci-dessous.

### Données générales

En l'absence de données réelles, le nombre de cartons par palette est estimé à 20 par défaut.

### Substances actives

La quantité de substance active est toujours disponible dans le RCP du médicament. En revanche, il est possible que le facteur d'émissions associé à cette substance active n'existe pas encore dans la base de données de l'outil. Dans ce cas, une méthodologie a été élaborée pour proposer une approximation pertinente (voir partie « Facteurs d'émission & Sources > Substances actives > Méthodologie en cas de FE inexistant »).

### Excipients

Quand la quantité d'excipient n'est pas disponible, une méthodologie d'estimation est proposée dans l'outil (Onglet « AIDE – Estim qtté Excipient »). Elle est différente pour les formes liquides et solides :

PROJET

- Formes liquides :
  - Pour chaque excipient dont la quantité est inconnue : la quantité d'excipient est estimée à 0,05 g / ml de médicament ;
  - Sauf pour les ajusteurs de pH (ex : acide chlorhydrique, hydroxyde de sodium) : 0,0005 g / ml de médicament.

- Formes sèches : La masse totale des excipients est estimée à partir de la masse des substances actives. En effet, d'après l'étude Reker D. 2018 [4], les excipients représentent 71% de la masse d'un comprimé. A cette masse totale d'excipient est soustraite la masse des excipients dont la quantité est connue.
  - Si le résultat est négatif, la quantité de chaque excipient par défaut est estimée à 0,5 g.
  - Sinon, la masse restante est répartie équitablement entre les excipients restants.

⇨ Ces valeurs par défaut ont été déterminées via la méthodologie suivante à l'aide des données actuellement disponibles.

L'estimation de la quantité d'excipient a été établie à 0,05 g/ml pour les formes liquides et les solutions. C'est une valeur inférieure aux doses seuil issues de la liste des excipients à effet notoire (environ 0,5 g/ml en s'appuyant sur les exemples cités ci-dessous) mais tout de même conservatrice et proche des concentrations de substance active. En effet, nous avons calculé une concentration moyenne de 0,025 g/ml en substance active sur une base d'une trentaine de médicaments liquides (voir tableur Excel – Annexe 1). Les doses seuils donnent un ordre de grandeur pour une quantité maximale d'excipient. Les valeurs réelles sont probablement bien inférieures (d'où le choix d'un ratio 10 fois inférieur).

Une exception a été prévue pour les ajusteurs de pH à 0,0005 g/ml, compte tenu des quantités de sodium et d'acide chlorhydrique détaillées dans le RCP du Lipidem (voir données précises ci-dessous).

Pour les formes sèches, l'estimation de 0,5 g a été déterminée en fonction des doses seuils par prise (ex : glycéril, PEF, mannitol etc. en s'appuyant sur les exemples cités ci-dessous). Cette valeur est utilisée uniquement en dernier recours si la méthodologie d'estimation à partir de la quantité de substance active donne un résultat incohérent (négatif).

Exemples de doses seuils issues de la liste des excipients à effet notoire

- Ex bronopol : 0,05% produit fini, soit 0,5 g/ml
- Formaldéhyde : 0,05% produit fini, soit 0,5 g/ml
- Paraformaldéhyde : 0,5% produit fini, soit 5 g/ml
- Ethanol : > 0,05 g / jour
- Glycéril : > 1g / prise
- PEG > 2 g /prise
- Sodium : > 200 mg/ jour
- Mannitol : > 1g/prise
- Xylitol : > 1 g/ prise

2 exemples de quantités d'excipients dévoilées dans le RCP du Lipidem 200mg/ml, émulsion pour perfusion

- NaOH : 2,6 mmol/L, soit 0,000104 g/ml
- HCl : 0,5 mmol/L, 0,000085 g/ml

Concernant les facteurs d'émissions des excipients, il est possible que le FE exact ne soit pas dans la base de données de l'outil. Dans ce cas, des approximations sont proposées et expliquées dans la partie « Facteurs d'émissions & sources > Excipients > Méthodologie en cas de FE inexistant ».

### Emballages


PROJET

Une estimation de l'empreinte carbone des emballages (matériaux et transformation) peut être calculée automatiquement par l'outil en sélectionnant l'option « par défaut ». Celle-ci s'appuie sur les informations logistiques / de conditionnement, la forme galénique et la quantité de médicament. Les calculs sont réalisés automatiquement dans l'onglet « Estimation emballages ». La méthodologie est présentée dans le tableur Excel - Annexe 2.

Annexe
2_Méthodologie_Estir

Quelques précisions méthodologiques générales :

- Les émissions de GES liées aux emballages et les quantités de matériaux (en grammes) sont ramenées à l'unité en fonction des données logistiques (nombre de DM par boîte, carton, palette).
- Les différents emballages (à l'exception des emballages individuels) sont toujours considérés comme des cubes par défaut (par soucis de simplification du modèle). Cela a tendance à minimiser la surface de l'emballage. Pour compenser cela ; nous avons considéré que les emballages occupaient à 100% l'espace du conditionnement supérieur.
- Les incertitudes des données d'activité sont par défaut fixées à 50% (considérées comme des estimations par défaut).

### Tableau récapitulatif des hypothèses de transport par défaut pour les médicaments

Les hypothèses par défaut utilisées pour le transport sont présentées dans le tableau ci-dessous. Les sources de ces hypothèses sont détaillées dans les paragraphes suivants.

| Etapes | Distances | Transport |
|---|---|---|
| Substances actives | **200 km** entre Zheijiang et Shanghai | Camion Asie |
| | **16 000 km** entre Shanghai et Bâle | Porte-conteneurs |
| | **40 km** de transport routier jusqu'à l'usine | Camion Europe |
| Excipients | **1 000 km** jusqu'à l'usine<br>(ex : entre usine ADM Saint Nolff et Bâle) | Camion Europe |
| Emballages | **500 km** des matériaux puis des emballages jusqu'au site de conditionnement | Camion Europe |
| Médicament | **250 km** jusqu'au site de conditionnement | Camion Europe |
| | **600 km** jusqu'à l'AGEPS | Camion Europe |
| | **30 km** entre l'AGEPS et l'hôpital | Camion France |

PROJET

### Transport amont de l'usine

#### *Transport substance active*

En l'absence d'information, les substances actives sont considérées produites en Asie et acheminées en Europe par porte-conteneur et transport routier.

Source origine des substances actives : Selon l'agence européenne du médicament, 80% [5] des fabricants de principes actifs pharmaceutiques pour les médicaments disponibles dans l'Union Européenne sont situés en dehors de l'Union (Chine et Inde). L'étude WHERE DO OUR ACTIVE PHARMACEUTICAL INGREDIENTS COME FROM ? – A WORLD MAP OF API PRODUCTION de Mundi Care et Pro Generika [6] montre une part légèrement plus importante des achats européens en provenance de la Chine (30%) que de l'Inde (27%). De plus, l'étude montre que les principes actifs sont principalement fabriqués dans la province de Zhejiang. C'est donc l'origine qui a été retenue par défaut dans l'outil.

Destination des substances actives et lieu de formulation des médicaments : Selon l'agence européenne du médicament, 40% [5] des médicaments finis commercialisés dans l'Union Européenne proviennent de l'étranger, et donc 60% de la production se fait en Europe. 68,3% des importations françaises de médicaments en 2022 proviennent de l'Union Européenne dont 14,8% pour l'Allemagne et 11,2% de la Suisse [7]. Les deux plus gros groupes pharmaceutiques européens Roche et Novartis ont leur siège à Bâle et leurs filiales allemandes dans la région frontalière suisse-allemande. Cette région a donc été retenue comme le lieu de production du médicament.

Route type retenue : le trajet type retenu est donc un transport de Zhejiang à Bâle soit :

- 200 km par camion entre Zhejiang et Shanghai (source : Google maps)
- ⇨ FE retenu : Transport en camion (dont parc, utilisation et infrastructure) (100%), RAS – Asie : 0,125 kgCO2e/t.km
- 16 000 km de transport maritime entre Shanghai et Bâle (source : Online Freight Shipping & Transit Time Calculator at Searates.com)
- ⇨ FE retenu : Porte-conteneur - Dry - Asie - Europe du Nord : 0,006 kgCO2e/t.km
- 40 km de transport routier jusqu'à l'usine.
- ⇨ FE retenu : Transport en camion (dont parc, utilisation et infrastructure) (100%), RAS – Asie : 0,125 kgCO2e/t.km

#### *Transport des excipients*

Il est difficile d'établir une route type pour les excipients car leur rôle et leur nature sont très variés. Les fournisseurs sont donc également très diversifiés et proviennent majoritairement d'autres secteurs d'activité (agroalimentaire, cosmétique, …) [8]. Dans le cadre de sa thèse, Camille Primard en liste un certain nombre [9]. Ces industries étant bien implantées en Europe ; nous avons choisi par défaut une localisation européenne pour la production des excipients. Pour calculer une route type, nous avons pris l'exemple du groupe ADM. Le groupe agro-alimentaire a 13 usines en France sur les 33 usines européennes de sa filière nutrition [10].

Route type retenue : transport routier entre l'usine ADM de Saint Nolff et Bâle, soit environ 1000 km (source : Google maps).

- ⇨ FE retenu : Transport en camion (dont parc, utilisation et infrastructure) (100%) – Europe : 0,09 kgCO2e/t.km

PROJET

*Transport des emballages*

Les valeurs prises par défaut en première approche proviennent des exemples de calculs d'empreinte carbone d'emballages dans l'outil BEE[2]. Cet outil a été développé par Citeo pour réaliser un bilan environnemental des emballages.

- Transport des matériaux jusqu'au site de production des emballages : 250 km.
- Transport des emballages jusqu'au site de conditionnement : 250 km.
- ⇨ FE retenu : Transport en camion (dont parc, utilisation et infrastructure) (100%) – Europe : 0,09 kgCO2e/t.km

### Transport aval de l'usine

*Transport du médicament jusqu'au site de conditionnement*

La valeur prise par défaut en première approche provient des exemples de calculs d'empreinte carbone d'emballages dans l'outil BEE[3]. Cet outil a été développé par Citeo pour réaliser un bilan environnemental des emballages.

- Transport du médicament jusqu'au site de conditionnement : 250 km.
- ⇨ FE retenu : Transport en camion (dont parc, utilisation et infrastructure) (100%) – Europe : 0,09 kgCO2e/t.km

*Transport du médicament entre l'usine et l'AGEPS*

Route type retenue : transport routier entre Bâle et l'AGEPS, soit 600 km (source : Google maps).

- ⇨ FE retenu : Transport en camion (dont parc, utilisation et infrastructure) (100%) – Europe : 0,09 kgCO2e/t.km

*Transport du médicament entre l'AGEPS et l'hôpital*

Le service Approvisionnement et Distribution (SAD) de l'Agence Générale des produits de santé (AGEPS) met à disposition les médicaments et certains produits de santé pharmaceutiques évalués puis référencés par l'autre service du pôle Pharmacie Hospitalière de l'AGEPS, pour l'ensemble des 37 établissements de l'AP-HP[4]. Cette Pharmacie à Usage Intérieur (PUI) stocke des produits de santé différents sur 2 sites distincts (situés rue Lavoisier à Nanterre). La distance moyenne entre le site de stockage de l'AGEPS et les hôpitaux a été calculée à environ 25 km, grâce à l'outil Google Maps (trajets en camion). Une hypothèse haute de 30 km a été retenue pour prendre en compte les éventuels problèmes de circulation, etc.

En première approche, les médicaments sont donc considérés acheminés par camion entre l'AGEPS et l'hôpital, avec une distance de transport par défaut de 30 km. Certains médicaments ne transitent pas par l'AGEPS mais nous avons retenu la situation majoritaire.

- ⇨ FE retenu : Transport en camion non spécifié France (dont parc, utilisation et infrastructure) (50%) : 0,270 kgCO2e/t.km

### Formulation et conditionnement

**Ratios énergétiques**

[2] https://bee.citeo.com/

[3] https://bee.citeo.com/

[4] Hors Hôpital maritime de Berck, Hôpital Paul Doumer, Hôpital marin de Hendaye, Hôpital San Salvadour et Hospitalisation à domicile.



PROJET

Lorsque les informations relatives à la formulation et au conditionnement du médicament sont inconnues, les hypothèses suivantes sont adoptées :

| Type | Energie | Ratios |
|---|---|---|
| Formes sèches | Electricité | 942 kWh / kg API |
| | Gaz naturel | 1 066 kWh PCI / kg API |
| Formes liquides | Electricité | 627 kWh / kg API |
| | Gaz naturel | 687 kWh PCI / kg API |
| Solutions | Electricité | 1,29 kWh / kg solution |
| | Gaz naturel | 0,03 kWh / kg solution |

Ces ratios ont été estimés à partir des BEGES ou rapports RSE de certains groupes pharmaceutiques français tels que Delpharm, Sanofi ou Fresenius. En effet, ces entreprises ont publié leurs consommations d'énergie, ou leurs émissions de gaz à effet de serre[5], pour répondre à leurs obligations règlementaires. La quantité de médicaments produite est également précisée dans leurs publications.

D'autre part, nous avons calculé la quantité moyenne de substance active par médicament sur une base d'une soixantaine de médicaments.

Ces deux sources nous ont permis de proposer des ratios énergétiques moyens en fonction de la quantité de substance active.

Nos ratios d'estimation se rapportent aux quantités de substance active et non au poids global du médicament car la masse des excipients fait pratiquement toujours l'objet d'une estimation et cela contribuerait à accroître encore l'incertitude. Pour les solutés, en revanche, les ratios sont calculés par rapport à la masse totale de soluté.

Les ratios comprennent aussi bien les consommations d'énergie liées à la production et que celles relatives au conditionnement car il est impossible de faire la distinction entre les deux avec cette méthodologie.

Les calculs et les sources sont détaillés dans les tableurs Excel - Annexes 3 et 4 :

Annexe 3_Méthodologie_Rati Annexe 4_Méthodologie_Rati

**Lieu de production**

En première approche, le médicament est considéré comme fabriqué à Bâle (voir Hypothèse & sources > Transport). Par conséquent, les facteurs d'émissions appliqués par défaut sont les suivants :

- Electricité - Europe OCDE : 0,218 kgCO2e/kWh
- Gaz naturel - Europe : 0,244 kgCO2e/kWh PCI

PROJET

[5] Ce qui nous permet indirectement de remonter à leurs consommations d'énergie.

### Pertes de matières durant la formulation

Un taux de 4,5% de pertes est pris en compte, en première approche.

Ce taux de pertes provient de l'outil ABPI Blister Pack Carbon Footprint Tool[6] :

- 2% de perte pendant le processus de formulation ;
- 0,5% pendant le conditionnement ;
- 2% de perte au moment de la distribution.

### Perte de matières des emballages

Un taux de 4,5% de perte d'emballages est pris en compte, en première approche.

Ce taux de pertes provient également de l'outil précédemment cité ABPI Blister Pack Carbon Footprint :

- 2,5% de perte pendant la production ;
- 2% de perte pendant la distribution.

### Fin de vie des emballages et notices

En l'absence d'information, les hypothèses à prendre en compte par défaut pour appliquer les facteurs d'émissions de fin de vie sont les suivantes :

| **HYPOTHESES** | **Flacon** | **Ampoule** | **Poche** | **Comprimé** | **Gélule** | **Sachet** | **Seringue** |
|---|---|---|---|---|---|---|---|
| Plastique | Emballages - Plastique rigide pétrosourcé PP autres emballages - Fin de vie moyenne filière | | Emballages - Plastique souple PE pétrosourcé - Fin de vie moyenne filière | Emballages - Plastique rigide pétrosourcé PP autres emballages - Fin de vie moyenne filière | Emballages - Plastique rigide pétrosourcé PP autres emballages - Fin de vie moyenne filière | Emballages - Plastique souple PE pétrosourcé - Fin de vie moyenne filière | Emballages - Plastique rigide pétrosourcé PP autres emballages - Fin de vie moyenne filière |
| Alu/inox | Emballages - Aluminium - Fin de vie moyenne filière | Emballages - Aluminium - Fin de vie moyenne filière | Emballages - Aluminium - Fin de vie moyenne filière | Emballages - Aluminium - Fin de vie moyenne filière | Emballages - Aluminium - Fin de vie moyenne filière | Emballages - Aluminium - Fin de vie moyenne filière | DAS - Incinération |
| Verre | Ordures ménagères résiduelles - Fin de vie moyenne | DAS - Incinération | | | | | DAS - Incinération |
| Carton | Emballages - Carton - Fin de vie moyenne filière | Emballages - Carton - Fin de vie moyenne filière | Emballages - Carton - Fin de vie moyenne filière | Emballages - Carton - Fin de vie moyenne filière | Emballages - Carton - Fin de vie moyenne filière | | Emballages - Carton - Fin de vie moyenne filière |
| Papier | | | | | | Emballages - Carton - Fin de vie moyenne filière | |

6 https://www.abpi.org.uk/media/1rdb3sno/carbon_blister_pack.xlsm

Dans le cas où l’utilisateur choisit l’option « par défaut » pour calculer les émissions liées aux emballages, les émissions liées à la fin de vie des emballages est également calculée automatiquement.

PROJET

# Calcul de l'empreinte carbone d'un dispositif médical

## Méthodologie globale

<u>Définition</u> : Un dispositif médical est un produit de santé destiné, par son fabricant, à être utilisé à des fins de diagnostic, de prévention, de contrôle, de traitement ou d'atténuation d'une maladie ou d'une blessure. [11]

La méthodologie de calcul de l'empreinte carbone d'un dispositif médical s'appuie sur les recommandations de la norme ISO 14040 et la méthode Bilan GES de l'ADEME. Elle est qualifiée d'approche « ACV simplifiée » car un cycle de vie standard et simplifié (voir schéma ci-dessous) a été modélisé. Le périmètre de cette ACV simplifiée est « du berceau à la tombe » (« cradle to grave »). Au sein de ce cycle de vie simplifié modélisé, l'utilisateur de l'outil doit paramétrer chaque étape avec les informations et données d'activité propres à un dispositif donné.

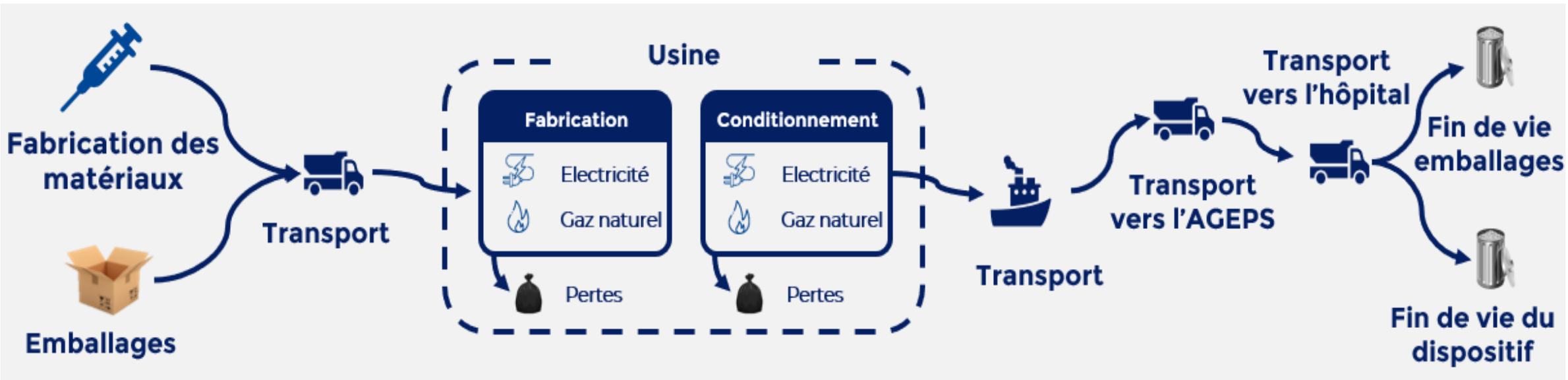


**Schéma du cycle de vie simplifié de la production et de l'utilisation d'un dispositif médical à l'AP-HP**

La méthodologie ACV nécessite de définir l'Unité Fonctionnelle avant de procéder aux calculs. La norme ISO 14044, définit l'Unité Fonctionnelle comme suit : "*Performance quantifiée d'un système de produits, destinée à être utilisée comme unité de référence dans une ACV*". Le rôle de l'Unité Fonctionnelle est de fournir une référence par rapport à laquelle les données d'activités seront définies et normalisées afin d'assurer la comparabilité des résultats de l'ACV sur une base commune.

Dans le cadre de cette méthodologie de calcul de l'empreinte carbone d'un DM, l'**Unité Fonctionnelle** est définie par défaut comme **la plus petite unité de dispositif médical possible** (ex : 1 aiguille pompeuse, 1 seringue 20 ml, 1 charlotte, etc.). Des précisions peuvent cependant éventuellement être apportées dans le nom / titre donné au calcul par l'utilisateur, en fonction du conditionnement du produit (1 paquet de 5 compresses stériles, 1 paire de surchaussures, etc.).

Les résultats des empreintes carbone de dispositifs médicaux ainsi calculées alimentent une base de données de « facteurs d'émissions », accessibles à tous les utilisateurs et pour tous les parcours de soins. Ces résultats sont accompagnés d'une incertitude également enregistrée dans la base (voir « Incertitude »).

## Matériel & informations nécessaires

La réalisation de l'empreinte carbone d'un dispositif médical nécessite de disposer :

- Des fiches techniques des produits, mentionnant les grammages et types de matériaux utilisés ou éventuellement juste les dimensions (voir méthodologie d'estimation dans Hypothèses & sources > Matériaux) ;
- Des informations logistiques sur le conditionnement du produit (nombre de DM par boîte, nombre de boîtes par carton, nombre de cartons par palette) ;

- ⇨ Au sein de l'AP-HP, ces données peuvent être retrouvées sur les sites du PIC Achat et de l'AGEPS :
    - o https://webacha.aphp.fr/accueil
    - o Marchés Equipements et Produits de santé (ap-hop-paris.fr)
- Optionnel : d'un échantillon de dispositif médical, afin de pouvoir réaliser des pesées si les fiches techniques ne sont pas accessibles ou sont incomplètes.

## Méthodologie détaillée

La méthodologie proposée repose sur un certain nombre de données d'activité, parfois difficiles à collecter. Si les données réelles sont disponibles / connues, il est conseillé de les saisir dans l'outil en remplacement des valeurs par défaut.

Sinon, des estimations sont proposées dans l'outil en première approche. Celles-ci sont présentées dans la partie « Hypothèses & sources ». Ces hypothèses sont fondées sur des sources explicitées également dans la partie « Hypothèses & sources ».

Pour chacun des postes ci-dessous, l'utilisateur doit saisir une donnée d'activité mais aussi sélectionner la méthodologie de collecte, c'est-à-dire la façon dont il a obtenu cette donnée. Cela permettra notamment de calculer l'incertitude des empreintes carbone ainsi estimées (voir « Incertitude »).

### Données générales

Cette catégorie permet de renseigner les informations générales du dispositif médical dont nous souhaitons calculer l'empreinte carbone :

- Nom du dispositif médical (voir recommandations « Nomenclature ») ;
- Stérile ? : préciser si le dispositif est stérile ou non ;
- Nombre d'utilisations (si usage unique, indiquer 1) ;
- Nombre d'unités dans une boîte ;
- Conditionnement 1 – Nombre de boîtes par carton ;
- Conditionnement 2 – Nombre de cartons par palette ;
- Référence fournisseur ;
- Numéro SAP (donnée interne à l'AP-HP) ;
- Nom du fabricant ;
- Responsable du calcul : Prénom et Nom de la personne en charge du calcul de cette empreinte carbone ;
- Fonction responsable : Fonction et organisation de la personne en charge du calcul ;
- Date du calcul.

### Matériaux

Les quantités de matériaux sont déterminées à partir des fiches techniques ou éventuellement en pesant le dispositif. La pesée est une méthodologie pas toujours précise et qui peut introduire un biais selon les caractéristiques de la balance utilisée. C'est pourquoi une méthodologie d'estimation des quantités est aussi proposée dans l'onglet « **AIDE – Masse Matériaux DM** » (voir Hypothèses & sources > Matériaux). Les quantités doivent être décomposées par type de matériaux :

- Plastique ;
- Métal ;
- Fibre ;
- Carton & autre ;

PROJET

- Composants électroniques.

Ces données sont saisies en grammes, à l'exception des composants électroniques, où l'unité doit être choisie en fonction du type de composant et du facteur d'émissions (plusieurs unités possibles : unité, kg, m ou m²).

### Emballages (matériaux et transformation)

Les impacts liés à la fabrication des emballages et de la notice du dispositif sont quantifiés en pesant ces éléments (grammes) ou en utilisant la méthodologie par défaut décrite dans la partie « Hypothèses & sources > Emballages ».

La masse des emballages doit être décomposée et renseignée en fonction des différents types de matériaux (carton, papier, aluminium, plastique, …). Elle est exprimée en grammes.

Le périmètre des emballages pris en compte comprend l'ensemble du conditionnement : de la palette jusqu'à la boîte de médicament. L'utilisateur précise dans l'outil si l'emballage concerne la palette, le carton, la boîte ou l'unité. Les formules de calcul permettent alors de ramener la part des émissions relatives à une unité de dispositif médical

Enfin, l'utilisateur choisit les facteurs d'émission correspondant aux matériaux des emballages. Il opte aussi dans le tableau suivant pour les facteurs d'émission relatifs à la transformation de ces emballages. Deux process de transformation maximum peuvent être sélectionnés par matériau.

### Transport amont de l'usine

#### *Transport des matériaux*

Ces trajets correspondent aux distances parcourues par les différents matériaux jusqu'à l'usine de production du dispositif médical.  Elles sont à renseigner en km par type de matériaux. Les émissions de GES associées sont calculées dans l'outil en fonction de cette distance et du poids des matériaux utilisés.

#### *Transport des emballages*

Ces transports correspondent :

- D'une part au trajet des matériaux d'emballage jusqu'au site de production des emballages ;
- D'autre part au trajet des emballages jusqu'au site de conditionnement du dispositif médical.

Ces distances sont exprimées en km. Les émissions de GES associées sont calculées dans l'outil en fonction de cette distance et du poids des matériaux d'emballage.

Lorsque ces informations ne sont pas accessibles, des estimations sont proposées dans la partie « Hypothèses & sources > Transport amont »

### Transport aval de l'usine

L'outil découpe en trois étapes le transport aval de l'usine :

- Transport du dispositif médical jusqu'au son site de conditionnement. Si celui-ci est le même que l'usine de production, le distance peut être de 0 km.
- Transport du dispositif médical du site de conditionnement jusqu'à la centrale d'achat (l'AGEPS pour l'AP-HP) si pertinent.
- Transport du dispositif médical depuis la centrale d'achat jusqu'à l'hôpital.

PROJET

⇨ Si le dispositif ne transite pas par la centrale d'achat, une seule des deux dernières lignes peut être complétée.

Ces distances sont exprimées en km. Les émissions de GES associées sont calculées dans l'outil en fonction de cette distance et du poids total du dispositif (somme des grammages des différents matériaux et des emballages).

Pour calculer les distances de transport, nous recommandons les outils suivants :

- Pour les trajets en bateaux : Online Freight Shipping & Transit Time Calculator at Searates.com
- Pour les transports routiers : Google Maps

Lorsque ces informations ne sont pas accessibles, des estimations sont proposées dans la partie « Hypothèses & sources > Transport aval »

### Processus de fabrication

Ce poste d'émission correspond aux consommations d'énergie (électricité, gaz,…) de l'usine de fabrication du dispositif médical, exprimées en kWh (kWh PCI pour le gaz naturel) / kg de matériaux.

Les consommations d'énergie sont à renseigner pour chaque type de matériaux. En cas de données indisponibles, des ratios de consommation par défaut sont proposés dans la partie « Hypothèses & sources > Processus de fabrication ».

### Processus d'emballage

Ce poste d'émission correspond aux consommations d'énergie (électricité, gaz,…) du site de conditionnement, exprimées en kWh (kWh PCI pour le gaz naturel) / kg d'emballage.

Des ratios par défaut sont proposés dans la partie « Hypothèses & sources > Processus d'emballage ».

### Stérilisation

Si le DM est stérile, les informations suivantes sont à renseigner dans l'outil :

- La capacité du stérilisateur : le nombre de palettes Euro qu'il peut contenir ;
- La durée du cycle de stérilisation en heures ;
- La puissance électrique du stérilisateur en Watt ;
- La concentration moyenne en oxyde d'éthylène sur la durée du cycle en g/L.

La consommation d'électricité requise par le stérilisateur est estimée à partir de la puissance électrique et de la durée du cycle. Elle est ramenée à une unité fonctionnelle grâce aux données relatives à la capacité du stérilisateur et aux informations logistiques (nombre de DM par boîte, nombre de boîtes par carton, nombre de cartons par palette).

Les émissions fugitives d'oxyde d'éthylène sont estimées à partir de la concentration en oxyde d'éthylène et du volume de la chambre du stérilisateur. Les hypothèses suivantes sont utilisées pour calculer le volume de la chambre :

| Nombre de palettes Euro | Volume de la chambre ($m^3$) |
|---|---|
| 4 | 12,7 |
| 8 | 24,8 |
| 10 | 30,6 |
| 12 | 36,9 |
| 16 | 49 |

PROJET

| | |
|---|---|
| 24 | 71,8 |
| 32 | 95 |

Source : Solutions de stérilisation par ETO, RSD [12]

A noter que la méthodologie de l'outil ne permet d'estimer que l'impact d'une stérilisation à l'oxyde d'éthylène. Les autres types de stérilisation ne sont pour l'instant pas pris en charge.

Des valeurs par défaut sont proposées dans la partie « Hypothèses & Sources > Stérilisation ».

### Pertes de matières des emballages

Lors du processus d'emballage, une partie des matériaux (carton, papier, aluminium, plastique, …) passe en perte ou rebus. Cette perte est exprimée en %.

Des hypothèses de pourcentages de pertes sont disponibles dans « Hypothèses & sources > Pertes ».

### Fin de vie

#### *Fin de vie du dispositif médical*

Les impacts liés à la fin de vie du dispositif médical sont quantifiés en choisissant le mode de traitement de ce déchet : recyclage, stockage, incinération, fin de vie filière.

#### *Fin de vie des emballages et notice*

Les impacts liés à la fin de vie des emballages et de la notice du médicament sont quantifiés en choisissant le mode de traitement de ces déchets : recyclage, stockage, incinération, fin de vie filière.

### Empreinte carbone totale

L'empreinte carbone totale du dispositif médical correspond à la somme des émissions de GES calculées dans chacun des postes listés ci-dessus. Un « facteur d'émission » est alors calculé est pour ce dispositif. Il peut être enregistré dans la base de données afin d'être utilisé dans le calcul de l'empreinte carbone d'un acte ou d'un parcours patient (voir protocole d'archivage).

### Emissions évitées dues à la fin de vie

Les émissions évitées sont calculées de manière séparée de l'empreinte carbone et sont issues de la valorisation matière et énergie des déchets (matériaux et emballages). Elles correspondent aux émissions GES qui ont été évitées grâce à la non production de matière vierge et/ou à la non production d'énergie conventionnelle. Ces émissions évitées n'impactent pas le facteur d'émission du dispositif médical.

## Hypothèses & sources

Un certain nombre d'hypothèses sont prises en compte dans cette méthode générale (distance de transport, ratios de consommations énergétiques par kg de matière ou d'emballage…). Il est conseillé d'ajuster ces estimations dans l'outil avec les données réelles du cas étudié quand celles-ci sont

PROJET

connues.

### Matériaux

Lorsque les masses des différents matériaux composant le dispositif médical ne sont pas précisées dans la documentation du fournisseur ou les fiches techniques, plusieurs options sont envisageables pour estimer la donnée :

- Le dispositif peut être pesé avec une balance ;
- Les quantités de matériaux peuvent être estimées à l'aide de l'onglet « AIDE – Masse matériaux DM ».

Les estimations de l'onglet « **AIDE – Masse matériaux DM »** reposent sur la méthodologie suivante :

- Chaque composant du DM est ramené à une forme simple : pavé (plein ou creux), cylindre (plein ou creux) ou sphère (pleine ou creuse) ;
- Le volume du composant est estimé à partir de la formule du volume de la forme correspondante :
    - Pavé droit = L × l × h (avec L la longueur, l la largeur et h la hauteur du pavé droit)
    - Cylindre = $\pi \times r^2 \times h$ (avec r le rayon et h la hauteur du cylindre)
    - Sphère = $4/3 \times \pi \times r^3$ (avec r le rayon de la sphère)
- La masse du composant est obtenue en multipliant ce volume par la masse volumique du matériau (voir tableau ci-dessous) ;
- Pour les **versions creuses** de ces formes, le volume de la forme « vide » est soustrait au volume de la forme pleine.

Les masses volumiques et leurs sources sont détaillées dans le tableau suivant :

PROJET

## Emballages

Une estimation de l'empreinte carbone des emballages (matériaux et transformation) peut être calculée automatiquement par l'outil en sélectionnant l'option « par défaut ». Celle-ci s'appuie sur les

| **Matériaux** | **Masse volumique (kg/m³)** | **Source** | **Lien** |
|---|---|---|---|
| PEBD | 927,5 | Eco-profiles of the European Plastics Industry_ PlasticsEurope 2005 | http://www.inference.org.uk/sustainable/LCA/elcd/external_docs/ldpe_311147f4-fabd-11da-974d-0800200c9a66.pdf |
| PEBBD | 920 | | |
| PEHD | 950 | | |
| PP | 910 | | |
| PS | 1040 | Matières plastiques -MS.LP - Académie de Dijon | http://mslp.ac-dijon.fr/IMG/pdf/matieres_plastiques.pdf |
| PSE | 30 | Polystyrène expansé ou PSE_Techniques de l'ingénieur_2008 | https://www.techniques-ingenieur.fr/base-documentaire/materiaux-th11/matieres-thermoplastiques-monographies-42147210/polystyrene-expanse-ou-pse-am3341/proprietes-du-polystyrene-expanse-am3341niv10004.html |
| PVC | 1380 | Wikipédia | https://fr.wikipedia.org/wiki/Polychlorure_de_vinyle |
| Polyuréthane | 1100 | Matières plastiques -MS.LP - Académie de Dijon | http://mslp.ac-dijon.fr/IMG/pdf/matieres_plastiques.pdf |
| Polycarbonate | 1200 | | |
| Acier inox | 7850 | Propriétés mécaniques des certains matériaux / Daniel GAY, Jacques GAMBELIN, "Dimensionnement des structures",édition Hermès 1999 | https://www.enib.fr/~mecatro/edm/S4/2021P/propri%C3%A9t%C3%A9s_m%C3%A9caniques_de_certains_mat%C3%A9riaux_1.pdf |
| Alliages d'aluminium | 2800 | | |
| Fibres de cellulose | 1500 | INRS - Fibres de cellulose - Fiche toxicologique n°282 | file:///C:/Users/4210122/Downloads/FicheTox_282.pdf |
| Silicone | 525 | Moyenne masses volumiques Elastomères de silicone - MEREFSA | https://www.merefsa.com/fr/produits/elastomere-de-silicone_kgr/ |
| Coton | 448 | DAGRIS - Dictionnaire du coton Densité universelle | http://hubrural.org/IMG/pdf/dagris_dictionnaire_coton.pdf |

informations logistiques et de conditionnement. Les calculs sont réalisés automatiquement dans l'onglet « Estimation emballages ». La méthodologie est présentée dans le tableur Excel - Annexe 2.

Annexe
2_Méthodologie_Estir

Quelques précisions méthodologiques générales :

- Les émissions de GES liées aux emballages et les quantités de matériaux (en grammes) sont ramenées à l'unité en fonction des données logistiques (nombre de DM par boîte, carton, palette).
- Les différents emballages (à l'exception des emballages individuels) sont toujours considérés comme des cubes par défaut (par soucis de simplification du modèle). Cela a tendance à minimiser la surface de l'emballage. Pour compenser cela, nous avons considéré que les emballages occupaient à 100% l'espace du conditionnement supérieur.

- Les incertitudes des données d'activité sont par défaut fixées à 50% (considérées comme des estimations par défaut).

### Tableau récapitulatif des hypothèses de transport par défaut pour les médicaments

| Etapes | Distances | Transport |
|---|---|---|
| Matériaux | **250 km** entre l'usine de fabrication des matériaux et l'usine (plastiques, métaux, fibres, carton, composants électroniques) | Camion Asie |
| Emballages | **500 km** des matériaux puis des emballages jusqu'au site de conditionnement | Camion Asie |
| Dispositifs médicaux | **250 km** jusqu'au site de conditionnement | Camion Asie |
| | **250 km** de l'usine en Chine jusqu'au port | Camion Asie |
| | **19 000 km** entre Shanghai et Plailly | Porte-conteneurs |
| | **45 km** jusqu'à l'AGEPS | Camion Europe |
| | **30 km** entre l'AGEPS et l'hôpital | Camion France |

DRAFT

Les sources de ces hypothèses sont détaillées dans les paragraphes suivants.

### Transport amont de l'usine

Concernant le transport en amont de l'usine, les valeurs prises par défaut en première approche proviennent des exemples de calculs d'empreinte carbone d'emballages dans l'outil BEE[7] développé par Citeo pour réaliser un bilan environnemental des emballages.

#### *Transport des matériaux*

En première approche, les matériaux sont considérés produits à une distance par défaut de 250 km de l'usine et sont considérés acheminés par camion.

⇨ FE retenu : Transport en camion (dont parc, utilisation et infrastructure) (100%), RAS – Asie : 0,125 kgCO2e/t.km

#### *Transport des emballages et notice*

- Transport des matériaux jusqu'au site de production des emballages : 250 km.
- Transport des emballages jusqu'au site de conditionnement : 250 km.

⇨ FE retenu : Transport en camion (dont parc, utilisation et infrastructure) (100%), RAS – Asie : 0,125 kgCO2e/t.km

[7] https://bee.citeo.com/

## Transport aval de l'usine

### *Transport du dispositif médical entre l'usine et le site de conditionnement*

Les valeurs prises par défaut en première approche proviennent des exemples de calculs d'empreinte carbone d'emballage dans l'outil BEE développé par Citeo pour réaliser un bilan environnemental des emballages. Cette distance est estimée à 250 km.

- ⇨ FE retenu : Transport en camion (dont parc, utilisation et infrastructure) (100%), RAS – Asie : 0,125 kgCO2e/t.km

### *Transport du dispositif médical entre le site de conditionnement et l'AGEPS*

En première approche, le dispositif médical est considéré comme fabriqué en Chine, à China Medical City. D'après une analyse de marché de BQ+ [13], China Medical City, dans la province de Jiangsu, est l'un des plus gros parcs industriels pharmaceutiques chinois en termes de nombres d'entreprises, avec plus de 110 fabricants de dispositifs médicaux. Par conséquent, la route type choisie est un trajet entre China Medical City et l'AGEPS. Les modes de transport et distances choisis par défaut sont :

- 250 km par camion entre China Medical City et Shanghai (source : Google maps)
- ⇨ FE retenu : Transport en camion (dont parc, utilisation et infrastructure) (100%), RAS – Asie : 0,125 kgCO2e/t.km
- 19 000 km de transport maritime entre Shanghai et Plailly (source : Online Freight Shipping & Transit Time Calculator at Searates.com)
- ⇨ FE retenu : Porte-conteneur - Dry - Asie - Europe du Nord : 0,006 kgCO2e/t.km
- 45 km de Plailly à l'AGEPS (source : Google maps)
- ⇨ FE retenu : Transport en camion (dont parc, utilisation et infrastructure) (100%) – Europe : 0,09 kgCO2e/t.km

### *Transport du dispositif médical entre l'AGEPS et l'hôpital*

En première approche, le dispositif médical est considéré acheminé par camion (distance de transport par défaut : 30 km). La distance moyenne entre l'AGEPS et les hôpitaux de l'AP-HP a été calculée (voir dans la partie Calcul de l'empreinte carbone d'un médicament > Hypothèses est sources > Transport aval). Même si la plupart des dispositifs médicaux ne transitent pas par l'AGEPS, cette estimation reste valable en ordre de grandeur et cette étape est non significative au regard du calcul global des émissions de GES.

- ⇨ FE retenu : Transport en camion non spécifié France (dont parc, utilisation et infrastructure) (50%) : 0,270 kgCO2e/t.km

## Processus de fabrication

Lorsque les informations relatives à la formulation et au conditionnement du médicament sont inconnues, les hypothèses suivantes sont adoptées :

**Ratios énergétiques**

| Type | Energie | Ratios |
|---|---|---|
| Matériaux plastiques | Electricité | 4,17 kWh / kg |
| | Gaz naturel | 2,35 kWh PCI / kg |
| Matériaux cartonnés | Electricité | 2,08 kWh / kg |

| | | |
|---|---|---|
| | Gaz naturel | 1,17 kWh PCI / kg |
| Matériaux fibrés | Electricité | - UU : 6,04 kWh / kg<br>- Réutilisable : 295,84 kWh / kg |
| | Gaz naturel | - UU : 3,40 kWh / kg<br>- Réutilisable : 166,63 kWh / kg |
| Matériaux métalliques | Electricité | 5,73 kWh / kg |
| | Gaz naturel | 3,22 kWh PCI / kg |
| Composants électroniques | Electricité | 0 kWh / kg |
| | Gaz naturel | 0 kWh PCI / kg |

Ces ratios ont été estimés à partir de facteurs d'émission « Mise en forme » de la base Empreinte de l'ADEME ou d'études scientifiques de type Analyse de Cycle de Vie. Les calculs et les sources sont détaillés dans le tableur Excel - Annexe 5 :

Annexe
5_Méthodologie_Ratio

PROJET

**Lieu de fabrication**

En première approche, le dispositif médical est considéré comme fabriqué en Chine, à China Medical City. En effet, d'après une analyse de marché de BQ+ [12], China Medical City, dans la province de Jiangsu, est l'un des plus gros parcs industriels pharmaceutiques chinois en termes de nombres d'entreprises, avec plus de 110 fabricants de dispositifs médicaux. Par conséquent, les facteurs d'émission appliqués par défaut sont les suivants :

- Electricité - Chine (République Populaire de Chine) : 0,614 kgCO2e / kWh
- Gaz naturel - Europe : 0,244 kgCO2e / kWh PCI[8]

### Processus d'emballage

Afin de déterminer les consommations d'énergie relatives au processus de conditionnement des dispositifs médicaux, deux ratios sont utilisés :

- Le premier ratio concernant l'électricité est de 0,739 kWh / kg d'emballage.
- Le second concernant le gaz naturel est de 0,416 kWh PCI / kg d'emballage.

Ces ratios ont été estimés à partir de facteurs d'émission « Transformation » collectés via l'outil BEE Citeo v 3.04. Les calculs et les sources sont détaillés dans le tableur Excel - Annexe 6 :

[8] Choix du FE « Europe », en l'absence de données plus précises disponibles.

Annexe
6_Méthodologie_Rati

**<u>Lieu de conditionnement</u>**

Comme pour la fabrication, le dispositif médical est considéré comme conditionné en Chine par défaut. Par conséquent, les facteurs d'émission appliqués par défaut sont les suivants :

- Electricité - Chine (République Populaire de Chine) : 0,614 kgCO2e / kWh
- Gaz naturel - Europe : 0,244 kgCO2e / kWh PCI[9]

### Stérilisation

En l'absence de données réelles, les données suivantes sont utilisées :

- Capacité de stérilisateur : 16 palettes Euro
- Durée du cycle totale de stérilisation : 12 heures
- Puissance du stérilisateur : 29 000 Watt
- Oxyde d'éthylène : 1 g/L

⇨ <u>*Source*</u> : Exemple d'un stérilisateur en acier inoxydable (Annexe 7)

Annexe 7_ Exemple
caractéristiques techn

Ces données sont extrêmement approximatives. En particulier, la quantité d'oxyde d'éthylène dépend des conditions de température et de pression. L'incertitude a été automatiquement forcée à 100%.

### Pertes de matières des emballages

En première approche, un taux de 4,5% de pertes de matériaux est pris en compte au niveau du processus de conditionnement.

Ce taux de pertes provient de l'outil ABPI Blister Pack Carbon Footprint Tool, cité dans la partie Méthodologie - Médicaments.

### Fin de vie

#### *Fin de vie des emballages et notice*

En l'absence d'information, les hypothèses à prendre en compte par défaut pour l'application des facteurs d'émission relatifs à la fin de vie des emballages sont détaillées dans le tableau suivant.

PROJET

[9] Choix du FE « Europe », en l'absence de données plus précises disponibles.

| HYPOTHESES | DM avec emballage bi-matière (papier+plastique souple) | DM avec emballage plastique rigide |
|---|---|---|
| Plastique | Emballages - Plastique souple PE pétrosourcé - Fin de vie moyenne filière | Emballages - Plastique rigide pétrosourcé PP autres emballages - Fin de vie moyenne filière |
| Carton | Emballages - Carton - Fin de vie moyenne filière | Emballages - Carton - Fin de vie moyenne filière |
| Papier | Emballages - Carton - Fin de vie moyenne filière | Emballages - Carton - Fin de vie moyenne filière |

## Incertitude

Dans sa « Méthode pour la réalisation des bilans de gaz à effet de serre conformément à l'article L.229-25 du code de l'environnement – Version 5 – 2020 », l'ADEME définit ainsi l'incertitude « paramètre associé au résultat d'une quantification qui caractérise la dispersion des valeurs pouvant être raisonnablement imputées à la grandeur quantifiée. L'incertitude mesure donc l'écart possible entre la valeur allouée à la grandeur quantifiée et sa valeur réelle. »

De nombreuses sources d'incertitude peuvent venir impacter la précision des calculs dans l'estimation d'une empreinte carbone. Dans cette méthodologie, il est considéré que l'incertitude est le résultat d'une combinaison des incertitudes des facteurs d'émission et des données sur les activités correspondantes.

<u>Incertitude liée au facteur d'émission</u>

Conformément aux différentes recommandations et bonnes pratiques diffusées par les standards internationaux, les émissions sont généralement estimées à l'aide de facteurs d'émission obtenus à partir de références et de bases de données institutionnelles. Ces facteurs auront été mesurés dans des circonstances particulières jugées typiques. Des incertitudes seront donc associées d'une part aux mesures d'origine, et d'autre part à l'utilisation des facteurs dans des circonstances autres que celles associées aux mesures d'origine. Par exemple, l'utilisation de facteurs d'émissions moyens x pour les excipients constitue une source importante d'incertitude puisqu'ils n'ont pas été calculés à l'origine dans ce but.

Les bases de données et sources d'émission fournissent généralement un niveau d'incertitude avec le facteur d'émission. Si cette information n'est pas fournie, l'incertitude est automatiquement estimée à 50%.

L'incertitude du facteur d'émission est automatiquement appliquée dans l'outil suite au choix du facteur d'émission par l'utilisateur.

<u>Incertitude liée aux données d'activité</u>

La fiabilité des estimations est aussi liée à la robustesse des données d'activité et à la manière dont elles ont été collectées. Certaines sont issues de mesures très précises et fournies par les fabricants des médicaments ou dispositifs médicaux. D'autres peuvent avoir fait l'objet d'estimations quand la donnée réelle n'était pas disponible. En s'appuyant sur les recommandations de la méthodologie de l'ADEME et de l'ABC, les règles suivantes ont été définies dans l'outil :

| Type de collecte | Incertitude |
|---|---|
| Fiche technique / donnée fournisseur | 5% |
| Pesée | 20% |
| Estimation par défaut | 40% |



PROJET

| | |
|---|---|
| Ratio de consommation | 100% |

Pour chaque poste d'émission, lorsque l'utilisateur saisit une donnée dans l'outil, il doit aussi sélectionner la manière dont il a récupéré cette information dans la colonne « Méthodologie collecte ». Le type de collecte « Estimation par défaut » fait référence notamment aux méthodologies proposées dans les onglets « AIDE- Estim qtté excipient » et « AIDE – Masse matériaux DM » et pour l'estimation des emballages par défaut. La collecte « ratio de consommation » renvoie principalement aux ratios utilisés pour estimer les consommations d'énergie relatives à la production et au conditionnement. En effet, ces estimations sont très approximatives car les données d'origine sont peu précises et les sources ne sont pas adaptées à chaque médicament et DM.

Combinaison des incertitudes et calcul de l'incertitude totale

Pour chaque poste d'émission, l'outil calcule une incertitude combinée et une incertitude combinée pondérée à l'aide des formules suivantes :

$\textbf{Incertitude combinée du poste} = \mathrm{c} = \sqrt{a^2 + b^2}$

$\text{Où } a = \text{incertitude liée au facteur d'émission}$

$\text{et } b = \text{incertitude liée à la donnée d'activité}$

$\textbf{Incertitude combinée pondérée} = \mathrm{i} = c \times D \div \sum D$

$\text{Où D} = \text{émission de GES du poste en gCO2e}$

$\text{Et } \sum D = \text{somme des émissions de tous les postes en gCO2e / empreinte carbone totale}$

L'incertitude totale correspond à la racine carrée des sommes des carrés des incertitudes de tous les postes et est donc calculée via la formule suivante :

$\textbf{Incertitude totale} = \mathrm{I} = \sqrt{\sum i^2}$

## Facteurs d'émissions & sources

La source et les autres facteurs sont présentés dans les paragraphes suivants.

### Substances actives

Afin d'utiliser correctement un facteur d'émission, il est nécessaire de connaître son périmètre. Dans le cadre de cette méthodologie, nous avons retenu des facteurs d'émission au format « Gate-to-Gate » dans la mesure des informations disponibles. Le format « Gate-to-Gate correspond uniquement aux émissions liées au processus du production. Les émissions de GES relatives à l'amont (matières premières, etc.) ou à l'aval de l'usine (transport, distribution, utilisation, fin de vie…) ne sont pas incluses dans le périmètre de ces facteurs d'émission.

Nous avons retenu ce format puisque l'amont et l'aval sont déjà pris en compte par ailleurs dans notre méthodologie.

#### *Liste des sources de FE utilisées*

Les sources pour les facteurs d'émission sont les suivantes :

PROJET

- Carbon footprint of the Austrian health sector - klima energie fonds

B670168-ACRP9-HealthFootprint-KR16AC0K13225-EB.pdf (klimafonds.gv.at)

⇨ Les facteurs d'émissions tirés de cette source ont été corrigés par la suite. En effet, les valeurs fournies concernent les émissions associées à la consommation énergétique du processus. La correction suivante : la part du processus dans les émissions totales est estimée à 10%. Cette hypothèse provient du rapport "Évaluation de l'empreinte carbone de l'ibuprofène" d'EcovaMed qui montre que le processus représente 10% des émissions de l'ibuprofène.

https://uploads-ssl.webflow.com/6151b650ce4cd9198b1fd7e8/6176e0cb1ac57152bc572efd_Ibuprofen_Carbon_footprint_Ecovamed_May_2021.pdf

- Des analyses de cycle de vie réalisées par les laboratoires pharmaceutiques dans le cadre des dossiers soumis aux autorités indiennes pour obtenir les autorisations environnementales nécessaires à leur activité :
    - SunPharma : Annexure-XVII – Life Cycle Analysis for M/s Sun Pharmaceutical Industries Limited (à partir de p 1043)
      DownloadPfdFile.aspx (environmentclearance.nic.in)
    - Solvay : Annexure-XVII – Life Cycle Analysis for M/s Solvay Specialities India Private Limited (p 925)

      DownloadPfdFile.aspx (environmentclearance.nic.in)
    - BEC Chemicals : ENVIRONMENTAL IMPACT ASSESSMENT(EIA) REPORT FORAN EXPANSION OF ACTIVE PHARMACEUTICAL INGREDIENT (API) MANUFACTURING UNIT by BEC Chemicals (p 146)
      DownloadPfdFile.aspx (environmentclearance.nic.in)

- ACS Sustainable Chemistry & Engineering [14] : onglet summary

  https://ndownloader.figstatic.com/files/14573810

- Combined simulationeoptimization methodology to reduce the environmental impact of pharmaceutical processes: application to the production of Penicillin V [15]

Combined simulation-optimization methodology to reduce the environmental impact of pharmaceutical processes: application to the production of Penicillin V | Elsevier Enhanced Reader

- Technical Potential for Energy and GWP Reduction in Chemical–Pharmaceutical Industry in Germany and EU—Focused on Biologics and Botanicals Manufacturing (Table 1 & 2)

Processes | Free Full-Text | Technical Potential for Energy and GWP Reduction in Chemical–Pharmaceutical Industry in Germany and EU—Focused on Biologics and Botanicals Manufacturing (mdpi.com)

- EcovaMed (ACV Juin 2022, fabrication en Chine – API Metformine) : https://uploads-ssl.webflow.com/6151b650ce4cd9198b1fd7e8/62c9be879749a0293c862949_METFORMIN_Life_Cycle_Assessment_ECOVAMED_June-2022.pdf

- EcovaMed (ACV Mai 2021 – API Ibuprofène) : https://uploads-ssl.webflow.com/6151b650ce4cd9198b1fd7e8/6176e0cb1ac57152bc572efd_Ibuprofen_Carbon_footprint_Ecovamed_May_2021.pdf

**Si le FE de l'API n'est pas encore dans la base de données** de l'outil, l'utilisateur peut choisir une estimation de facteur d'émission grâce à l'onglet « **AIDE – Choix FE API** ».

Les facteurs d'émissions moyens sont les suivants :

- **API – Faible** : 600 kgCO2e / kg API
- **API – Moyen** : 1500 kgCO2e / kg API
- **API – Elevé** : 7000 kgCO2e / kg API

Ces FE sont issus de l'outil ABPI Blister Pack Carbon Footprint Tool : https://www.abpi.org.uk/media/1rdb3sno/carbon_blister_pack.xlsm

Or, l'outil ABPI ne précise pas clairement comment choisir entre les trois facteurs. Une autre méthodologie de cet outil "calculated" permet d'estimer un FE en fonction de la chiralité de la molécule, du volume de production et du nombre d'étapes de la fabrication. Ces données étant rarement disponibles, nous avons proposé une méthodologie simplifiée permettant le choix du FE API. Celle-ci s'inspire de la méthodologie de l'outil ABPI et d'autres sources citées plus bas.

La méthodologie proposée dans l'onglet « AIDE – Choix FE API » repose sur la note moyenne entre 4 critères. Pour chaque API, ces critères sont évalués de 1 (consommations énergétiques et émissions de GES faibles) à 3 (consommations énergétiques et émissions des GES élevées). Le tableau suivant permet d'attribuer le FE API moyen en fonction de la note obtenue :

| | |
|---|---|
| Note moyenne > 2 | API - Elevé |
| 1,5 < Note moyenne =< 2 | API - Moyen |
| Note moyenne =< 1,5 | API - Faible |

- **Critère 1** : Complexité de la molécule

La structure chimique, le nombre et le types d'atomes ou de groupes fonctionnels d'une molécule ont une influence sur le processus de production de la substance active. Ceci va notamment agir sur le nombre d'étapes nécessaires à la fabrication, chacune pouvant exiger des conditions de réaction spécifiques et potentiellement énergivores. Des substances actives complexes de type polysaccharides, peptides ou protéines recombinantes se verront attribuées une note de 3 tandis que des molécules simples comme le paracétamol (un cycle benzénique substitué par un groupe hydroxyle et un amide) sont notées 1. La chiralité de la molécule contribue à augmenter la note.

- **Critère 2** : Origine de la substance active

Les différents types de fabrication impactent la consommation d'énergie nécessaire à la fabrication de l'API. La liste ci-dessous reprend les principaux types de fabrication (source : Annex 2, WHO Technical Report Series 957, 2010) et propose une note recommandée. Celle-ci a été déterminée en fonction du nombre d'étapes nécessaires et de leurs complexité (sources plus bas).

| Type fabrication | Note recommandée | Commentaires |
|---|---|---|
| Fabrication chimique | 2 | Les procédés de fabrication chimique peuvent varier en termes de consommation d'énergie en fonction de la complexité de la synthèse, des réactions exothermiques ou endothermiques impliquées et de l'efficacité des technologies utilisées. |
| Substances actives issues de sources animales | 3 | L'extraction de substances actives à partir de sources animales peut nécessiter des processus d'extraction complexes et intensifs en énergie, en particulier pour purifier les substances à partir de tissus biologiques. |



PROJET

| | | |
|---|---|---|
| Substances actives extraites de sources végétales | 2 | L'extraction de substances actives à partir de sources végétales peut nécessiter des étapes de traitement et de purification, mais on les suppose moins gourmandes en énergie que pour une source animale (exigences de sécurité moins fortes, matière première moins impactante, ...). |
| Extraits de plantes utilisés comme substance active | 1 | Ce type de fabrication comprend moins d'étapes et notamment pas de purification, ce qui suggère une consommation globalement moins importante en énergie. |
| Substances actives composées de plantes broyées ou pulvérisées | 1 | Ce type de fabrication comprend moins d'étapes et notamment pas de purification, ce qui suggère une consommation globalement moins importante en énergie. |
| Biotechnologie fermentation / culture cellulaire | 3 | Le process de production du biomédicament est très différent de celui du médicament chimique. Les consommations d'énergie comprennent notamment le fonctionnement des chambres froides, chaleur nécessaire à la production de vapeur et d'eau pharmaceutique, production de chaleur pour le milieu de culture (température entre 25 et 35°), milieu de culture stérile...[10] |
| Fermentation classique pour la production d'une substance active | 2 | La fermentation est un processus qui nécessite des conditions bien spécifiques de température et de pression mais c'est un procédé ancien et largement maîtrisé dans d'autres industries que le secteur pharmaceutique. |

- **Critère 3** : Conditions particulières

Certaines substances peuvent nécessiter des conditions de réaction spécifiques, telles que des températures ou pression élevées, qui peuvent entrainer une utilisation accrue d'énergie pour maintenir ces conditions. Certaines substances actives peuvent aussi être produites par des réactions exothermiques, c'est-à-dire des réactions dégageant de la chaleur. Le contrôle de la température et le refroidissement peuvent aussi entrainer une consommation d'énergie plus élevée. En cas d'absence d'information sur ce critère, une note de 2 est attribuée par défaut.

- **Critère 4** : Volumes de production

L'efficacité énergétique des processus de production est souvent proportionnelle aux volumes de production (économies d'échelle, investissement dans des technologies efficientes en énergie, etc.). Ainsi, des médicaments produits en quantité très importante (ex : paracétamol) auront une note de 1 tandis que des médicaments rares se verront attribuer une note de 3 sur ce critère. L'AP-HP s'est parfois appuyée sur ses propres données d'achat et d'utilisation de médicaments pour évaluer ce critère.

Sources utilisées :

- LEEM : 2022-12-16 - Rapport final Impact de la transition écologique sur les métiers et compétences des industries de santé
- Bonnes pratiques de fabrication pour les substances actives pharmaceutiques (Annex 2, WHO Technical Report Series 957, 2010)
- Chat GPT

Pour les « facteurs d'émission » médicament évalués par l'AP-HP, la liste des notes attribuées à chaque substance active et leurs justifications sont disponibles sur demande.

[10] Dans une prochaine version, il serait probablement pertinent de développer une méthodologie propre aux biotechnologies.



PROJET

## Excipients

Afin d'utiliser correctement un facteur d'émission, il est nécessaire de connaître son périmètre. Dans le cadre de cette méthodologie, nous avons retenu des facteurs d'émission au format « Gate-to-Gate » dans la mesure des informations disponibles. Le format « Gate-to-Gate correspond uniquement aux émissions liées au processus du production. Les émissions de GES relatives à l'amont (matières premières, etc.) ou à l'aval de l'usine (transport, distribution, utilisation, fin de vie…) ne sont pas incluses dans le périmètre de ces facteurs d'émission.

Nous avons retenu ce format puisque l'amont et l'aval sont déjà pris en compte par ailleurs dans notre méthodologie.

### *Liste des sources de FE utilisés*

Les sources pour les facteurs d'émission des excipients sont les suivantes :

- L'outil ABPI Blister Pack Carbon Footprint Tool – Onglet Original Excipient EFs :

https://www.abpi.org.uk/r-d-manufacturing/abpi-blister-pack-carbon-footprint-tool

- Base Carbone (version 22.0), ADEME

https://base-empreinte.ademe.fr/

- Inventaire des émissions de GES appliqué au secteur des Spiritueux au travers de la méthode Bilan Carbone® - GUIDE SECTORIEL – Carbone 4 – Juin 2011

https://librairie.ademe.fr/changement-climatique-et-energie/3545-inventaire-des-emissions-ges-applique-au-secteur-spiritueux-au-travers-de-la-methode-bilan-carbone.html

- Guide méthodologique pour la réalisation de Bilan Carbone® des entreprises de boulangerie, pâtisserie, viennoiserie industrielles – Janvier 2011 (pages 69 à 72)

https://librairie.ademe.fr/changement-climatique-et-energie/3345-guide-methodologique-pour-la-realisation-de-bilan-carbone-des-entreprises-de-boulangerie-patisserie-viennoiserie-industrielles.html

- Solvay : Annexure-XVII – Life Cycle Analysis for M/s Solvay Specialities India Private Limited (p 925)

- Guide méthodologique d'évaluation des émissions de gaz à effet de serre des services de l'eau et de l'assainissement – ASTEE – Mise à jour 2018

https://www.astee.org/publications/guide-methodologique-des-emissions-de-gaz-a-effet-de-serre-des-services-de-leau-et-de-lassainissement/

- The Carbon Footprint of Titanium Dioxide Pigment – December 2023 – Titatium Dioxyde Manufacturers Association

https://tdma.info/wp-content/uploads/2017/09/The-carbon-footprint-of-titanium-dioxide.pdf

- EcovaMed (ACV Juillet 2023, fabrication en Chine - PVP) : https://uploads-ssl.webflow.com/6151b650ce4cd9198b1fd7e8/64d00de2347c14534e5be458_PVP_Life_Cycle_Assessment_ECOVAMED_July-2023.pdf

PROJET

A ce jour, un facteur d'émission unique est proposé dans l'outil pour l'eau purifiée et l'eau pour préparations injectables. Il correspond à la moyenne des deux process de production de l'étude ci-dessous, soit 0,00155 kgCO2e / L.

- Yuan Yuan, Jiaqi Lu, Dungang Gu, Yuhang Lou, Na Xue, Guanghui Li, Wenjie Liao, Nan Zhang - Deduction of carbon footprint for membrane desalination processes towards carbon neutrality: A case study on electrodeionization for ultrapure water preparation, Desalination, Volume 559, 2023, 116648, ISSN 0011-9164

https://doi.org/10.1016/j.desal.2023.116648

Pour le cas particulier des anticorps monoclonaux, un calcul automatique permet d'estimer l'empreinte de l'eau pour préparations injectables. L'étude Cataldo, 2020 [16], propose un indicateur variant de 16 à 89 kgCO2e / kg d'anticorps monoclonaux. Pour l'outil, une moyenne de 52,5 kgCO2e / kg d'anticorps monoclonaux a été utilisée.

### *Méthodologie en cas de FE inexistant*

**Si le facteur d'émission exact n'est pas encore présent dans la base de données** de l'outil, des estimations sont proposées dans l'onglet « **AIDE – Choix FE Excipient** ».

Elles ont été construites à partir de moyennes de facteurs d'émissions de la base de données selon la nature et / ou le processus de fabrication de l'excipient.

| Catégorie Excipient | Moyenne FE (kg CO2e/ kg) |
|---|---|
| **Excipient - Chimie lourde, de base** | **4,20** |
| *Acides gras (acide oléique, acide stéarique,etc.)* | *23,66* |
| *Alcools (éthanol, glycol, glycérol, …)* | *1,21* |
| *Autre sucres (mannitol, carmelloses, croscarmelloses, hypromellose, …)* | *3,06* |
| *Cellulose et dérivés* | *3,74* |
| *Hydrocarbures (vaseline, paraffine, …)* | *0,83* |
| *Polyéthylènes-glycols (P.E.G, P.O.E.G, ou macrogols)* | *2,10* |
| *Povidone, crospovidone, PVP* | *21,70* |
| *Produits minéraux (silice, talc, oxyde de titane, …)* | *2,13* |
| *Composés minéraux de base* | *1,19* |
| **Excipient - Origine naturelle** | **7,96** |
| *Amidons et dérivés* | *1,43* |
| *Arômes d'origine naturelle* | *20,64* |
| *Gélatine* | *1,78* |
| *Glycérines et huiles végétales* | *12,62* |
| *Gommes* | *1,47* |
| *Oxydes de fer* | *0,03* |
| *Sucres et dérivés (saccharose, lactose, glucose, …)* | *1,78* |
| *Substances extraites d'algues (alginate de sodium, carraghénates, gélose, agar-agar,etc.)* | *6,77* |
| **Excipient - Chimie fine, de spécialité** | **231,09** |
| *Antioxydants aminiques et phénoliques* | *324,07* |
| *Arômes d'origine synthétique* | *9,21* |
| *Colorants d'origine synthétique* | *2,33* |

PROJET

| | |
|---|---|
| *Tensio-actifs anioniques (savons et dérivés sulfatés)* | *308,42* |
| *Tensio-actifs cationiques (halogénures d'ammonium quaternaires,…)* | *341,10* |

Ces moyennes ont permis de proposer des approximations pour des excipients de la même nature (cf tableau ci-dessus) mais aussi des estimations pour des catégories non couvertes dans la base de données :

| **Produits** | **Choix du FE (kgCO2e /kg)** | **Explications** |
|---|---|---|
| Cires et beurres - Estimation | 12,62 | Idem Huiles végétales |
| Glycérides hémi-synthétiques et synthétiques - Estimation | 23,66 | Idem Acides gras |
| Alcool gras (alcool céthylique, alcool stéarylique, …) - Estimation | 4,20 | Excipient - Chimie de base, lourde |
| Esters non glycéridés (polystates B, stéréates de PEG, cétiols, …) - Estimation | 231,09 | Excipient - Chimie fine, de spécialité |
| Silicones - Estimation | 231,09 | Excipient - Chimie fine, de spécialité |
| Tensio-actifs naturels (chlorestérol, lécithine, saponine, …) - Estimation | 7,96 | Excipient - Origine naturelle |
| Alkylolamine, alcanolamine, et éthanolamines - Estimation | 231,09 | Excipient - Chimie fine, de spécialité |
| Colorants d'origine naturelle - Estimation | 7,96 | Excipient - Origine naturelle |
| Acide aminé - Fermentation bactérienne - Estimation | 7,96 | Excipient - Origine naturelle |
| Acide aminé - Synthèse chimique - Estimation | 231,09 | Excipient - Chimie fine, de spécialité |
| Parabènes - Estimation | 231,09 | Excipient - Chimie fine, de spécialité |
| Excipient - Chimie fine, de spécialité - Estimation | 231,09 | Moyenne des FE connus (TCD) |
| Excipient - Chimie de base, lourde - Estimation | 4,20 | Moyenne des FE connus (TCD) |
| Excipient - Origine naturelle - Estimation | 7,96 | Moyenne des FE connus (TCD) |

PROJET

## Energie

Les sources des facteurs d'émissions sont :

- Base Carbone (version 22.0), ADEME

https://base-empreinte.ademe.fr/

- IEA - Emissions Factors 2022

https://www.iea.org/data-and-statistics/data-product/emissions-factors-2022

### Matériaux & Emballages

Les sources des facteurs d'émissions sont :

- Base Carbone (version 22.0), ADEME

https://base-empreinte.ademe.fr/

- Base Impacts, ADEME

https://base-empreinte.ademe.fr/

- PlasticsEurope

http://www.inference.org.uk/sustainable/LCA/elcd/external_docs/petb_31116f00-fabd-11da-974d-0800200c9a66.pdf

https://www.pedagogie.ac-aix-marseille.fr/upload/docs/application/pdf/2015-11/4-_eco-profile_pe_2014-04.pdf

- Guide des facteurs d'émissions du Bilan Carbone® Entreprises et Collectivités Version 6.1 édité par l'ADEME en juin 2010 :

http://23dd.fr/images/stories/Documents/PV/Ademe_Metro_Chapitre_2_Energie.pdf

- Silicon-chemistry carbon balance : an assessment of greenhous gas emissions and reductions

https://www.silicones.eu/wp-content/uploads/2019/05/SIL_exec-summary_en.pdf

- Ecoinvent (BEGES Bouygues 2015)

https://www.bouygues-tp.com/uploads/page/21/Rapport_BEGES_2015_BYTP_SA.pdf

- France Stratégie – Comment évaluer l'externalité carbone des métaux

https://www.strategie.gouv.fr/sites/strategie.gouv.fr/files/atoms/files/fs-2020-na96-externalite-carbone-metaux-octobre.pdf

- Buitrago-Tello, Rodrigo; Venditti, Richard A.; Jameel, Hasan; Yao, Yuan; Echeverria, Darlene (2022): Carbon Footprint of Bleached Softwood Fluff Pulp: Detailed Process Simulation and Environmental Life Cycle Assessment to Understand Carbon Emissions. ACS Publications. Collection.

https://doi.org/10.1021/acssuschemeng.2c00840

- Total Clarity - Diamond Producers Association - Rapport Trucost

https://collectif-diamant.fr/uploads/607e9d4f726d0_DPA_TotalClarity_Report_FR.pdf

- Les matériaux biosourcés – Arcanne – 07/04/2020 :

https://associationarcanne.files.wordpress.com/2020/04/arcanne-bs.2020.04.pdf

PROJET

Les facteurs d'émission correspondant aux process de transformation des matériaux d'emballage sont tirés de l'outil BEE Citeo v 3.04 :

https://bee.citeo.com/

### Réactifs et substances

- Labos 1 point 5

https://apps.labos1point5.org/purchases-simulator

- Formaldéhyde : Review and environmental footprint assessment of various formalin production pathways, Jan Puhar, Damjan Krajnc, Lidija Čuček, Annamaria Vujanović,

https://www.sciencedirect.com/science/article/pii/S0959652622041099

### Transport & déplacements

Les sources des facteurs d'émissions sont :

- Base Carbone (version 22.0), ADEME

https://base-empreinte.ademe.fr/

### Fin de vie

Les sources des facteurs d'émissions sont :

- Base Carbone (version 22.0), ADEME

https://base-empreinte.ademe.fr/

### Consommables & équipements annexes

Les sources des facteurs d'émissions sont issus de :

- Base Carbone (version 22.0), ADEME

https://base-empreinte.ademe.fr/

### Hygiène & Nettoyage

Les sources des facteurs d'émissions sont issus de :

- Base Carbone (version 22.0), ADEME

https://base-empreinte.ademe.fr/

PROJET

### Consommation d'eau

Eau de réseau (consommation et traitement) : 0,394 kgCO2e / m³

La source est la Base Carbone (version 22.0), ADEME et le FE est la somme de :

- Achats de biens > Eau, traitement et distribution d'eau : 0, 132 kgCO2e / m³
- Traitement des déchets > Eaux usées > eaux usées, Eau, eau potable, eau de réseau : 0,262 kgCO2e / m³

# Protocoles transversaux utilisés

## Nomenclature

Pour assurer la clarté et l'homogénéité des facteurs d'émissions calculés par cet outil, les paragraphes suivants décrivent la nomenclature adoptée. Les facteurs d'émissions sont tous intitulés en français, en minuscules avec des majuscules uniquement à la première lettre ou après un tiret.

### Médicaments

Idéalement, le libellé du FE est repris du libellé commercial du médicament tel que décrit sur le site de l'AGEPS.

http://informations-marches-ageps.ap-hop-paris.fr/index.php?id=6034e106c616d332ccf3b10b7d2577d0

Les informations obligatoires et l'ordre à privilégier sont les suivants :

Nom – Quantité [Unité] – Forme Galénique - CIP 13

La référence SAP, le fabricant, le nom du responsable du calcul, et la date sont à renseigner dans les données générales et seront ainsi enregistrées dans les colonnes dédiées de la base de données des FE.

### Dispositif médical

Idéalement, le libellé du FE est repris du libellé commercial du médicament tel que décrit sur le site de l'AGEPS ou d'ACHAT

http://informations-marches-ageps.ap-hop-paris.fr/index.php?id=6034e106c616d332ccf3b10b7d2577d0

https://webacha.aphp.fr/accueil

Les informations obligatoires et l'ordre à privilégier sont les suivants :

Nom – Taille [Unité] - Référence fournisseur

La référence SAP, le fabricant, le nom du responsable du calcul, et la date sont à renseigner dans les données générales et seront ainsi enregistrées dans les colonnes dédiées de la base de données des FE.

PROJET

## Archivage

L'enregistrement des facteurs d'émissions en vue de leur réutilisation dans le cadre du calcul de l'empreinte carbone d'un parcours patient est réalisé à deux niveaux :

- L'outil permet de copier-coller la ligne du facteur d'émission (acte, médicament, dispositif médical) calculé et de l'insérer dans la base de données des facteurs d'émissions. Celui-ci est alors enregistré et disponible dans la nouvelle version de l'outil. Le nom du responsable du calcul, la date, le fabricant et la référence SAP sont tracés.
- Le ficher de calcul de ce facteur d'émission est conservé et enregistré sur le réseau partagé de l'AP-HP, de sorte que les hypothèses de calcul et les données d'entrée peuvent être à tout moment retrouvées / modifiées / mises à jour.

# Acronymes

| **Acronymes** | **Définition** |
|---|---|
| API | Active Principal Ingredient ou substance active |
| ACV | Analyse de Cycle de Vie |
| $CO_2$ | Dioxyde de carbone |
| $CO_2e$ | Équivalent dioxyde de carbone |
| DM | Dispositif médical |
| FE | Facteur d'émission |
| GES | Gaz à effet de serre |
| PRG | Pouvoir de réchauffement global |

PROJET

## Bibliographie


[1] Ministère de la Santé et de la Prévention, «Qu'est-ce qu'un médicament ?,» 16 03 2022. [En ligne]. Available: https://sante.gouv.fr/soins-et-maladies/medicaments/le-bon-usage-des-medicaments/article/qu-est-ce-qu-un-medicament. [Accès le Avril 2023].

[2] Ministère de la Santé et de la Prévention, «Principe actif ou substance active,» 13 05 2016. [En ligne]. Available: https://sante.gouv.fr/soins-et-maladies/medicaments/glossaire/article/principe-actif-ou-substance-active. [Accès le Avril 2023].

[3] Ministère de la Santé et de la Prévention, «Excipient,» 13 05 2016. [En ligne]. Available: https://sante.gouv.fr/soins-et-maladies/medicaments/glossaire/article/excipient. [Accès le Avril 2023].

[4] B. S. S. C. A. K. S. J. F. J. T. G. Reker D, « "Inactive" ingredients in oral medications. Sci Transl Med. 2019 Mar 13;11(483):eaau6753. doi: 10.1126/scitranslmed.aau6753. PMID: 30867323; PMCID: PMC7122736.».

[5] European Medicines Agency, «European and US regulators agree on mutual recognition of inspections of medicines manufacturers,» Press release 02/03/2017.

[6] Progenerika, «Where do our active pharmaceutical ingredients come from ? - A world map of API production - Final report,» Berlin, 2020.

[7] LEEM, «Bilan économique - Edition 2022».

[8] A. Dureuil, «Un marché difficile à réglementer,» *L'Usine Nouvelle,* 2010.

[9] C. Primard, «Mise en place du guideline excipient dans un établissement pharmaceutique de recherche & développement,» 2018.

[10] ADM, «Scaling Impact - 2021 Corporate Sustainability Report,» 2021.

[11] Ministère de l'Economie, des Finances, et de la Souveraineté Industrielle et Numérique, «Dispositifs médicaux,» 24 11 2021. [En ligne]. Available: https://www.economie.gouv.fr/dgccrf/Publications/Vie-pratique/Fiches-pratiques/Dispositifs-medicaux. [Accès le Avril 2023].

[12] RSD, «Solutions de stérilisation par ETO,» [En ligne]. Available: https://www.rsd-engineering.com/uploads/images/RSD%20-%20Equipements%20de%20st%C3%A9rilisation%20ETO%20catalogue.pdf.

[13] BQ+, «Market analysis of Medical Devices in China,» 17 Mai 2022. [En ligne]. Available: https://bq-md.com/market-analysis-of-medical-devices-in-china/#elementor-action%3Aaction%3Dpopup%3Aopen%26settings%3DeyJpZCI6IjcyOCIsInRvZ2dsZSI6ZmFsc2V9.

[14] H. T. J. D. S. P. C. P. A. J. B. Z. M. J. E. Abhijeet G. Parvatker, «Cradle-to-gate Greenhouse Gas Emissions for Twenty Anesthetic Active Pharmaceutical Ingredients based on Process Scale-up and Process Design Calculations».

[15] G. G.-G. L. J. Robert Brunet, «Combined simulationeoptimization methodology to reduce the environmental impact of pharmaceutical processes: application to the production of Penicillin V,» *Journal of Cleaner Production,* 2014.


PROJET

[16] B. S. K. M. K. B. O. S. M. L. A. J. P. S. Alessandro Luigi Cataldo, «Water related impact of energy: Cost and carbon footprint analysis of water for biopharmaceuticals from tap to waste,» *Chemical Engineering Science: X,* vol. 8, 26 Septembre 2020.

[17] ProCarton, «Glossaire de l'industrie du carton plat et des cartonnages,» 2008.

[18] hellopro.gr, «Tout savoir sur les palettes,» 07 111 2022. [En ligne]. Available: https://conseils.hellopro.fr/tout-savoir-sur-les-palettes-1295.html. [Accès le Avril 2023].

[19] VereinigtePapierwarenfabriken, «Spécification du produit : Sachets ou gaines transparents qualité MM70-1/PP50-1,» 2019.

[20] Valipac, «Panorama des poids de référence des emballages industriels,» 2019.

PROJET
