# Supplementary material for: Intravenous-to-enteral drug substitution in the ICU: a single-centre analysis of the potential to reduce the environmental footprint of critical care
Source: Crit Care. 2026 Sep 22;30:487. doi: 10.1186/s13054-026-06331-z (PMC13599322; doi:10.1186/s13054-026-06331-z)
Supplement: Supplementary file 2 [file 13054_2026_6331_MOESM1_ESM.docx]

**Supplementary data 1**

**Methods**
*Study design and setting:* We conducted a retrospective observational study across eight medical and surgical ICUs within a French university-affiliated hospital network. These units included four medical ICUs (general medical, neurological, hepatogastroenterology, and cardiac) and four surgical ICUs (general surgical, neurosurgical hepatobiliary and cardiovascular surgical), representing 186 beds, and caring for a wide range of critically ill patients.

*Data collection and medication selection:* We extracted the list of all IV medications dispensed throughout 2024. Data were extracted from the hospital pharmacy information system Copilote (Docaposte, France), which manages medication supply, dispensing, and traceability within the institution. Structured queries were developed using the built-in reporting module to retrieve relevant variables (e.g., dispensing data, timestamps, care units). Data were anonymized prior to analysis in compliance with applicable data protection regulations. A consolidated inventory of IV medications, using international nonproprietary name used across all units was then established.

*Assessment of enteral feasibility:* Medications with an available oral formulation were identified using Copilote (Docaposte, France). Oral formulations included syrups, tablets, capsules, oral suspensions and effervescent forms. For each drug, feasibility of administration via a nasogastric or orogastric tube was assessed based on pharmaceutical formulation (e.g., crushable tablet, disintegrating tablet, oral suspension, syrup, pellets, openable capsule) according to the national list of crushable medications developed by *OMéDIT Normandie* in collaboration with the French Society of Clinical Pharmacy (supplementary data)**.** This reference database compiles information derived from summary of product characteristics and data provided by pharmaceutical manufacturers. When necessary, additional sources cited within the database (e.g., *Thériaque*, Geneva University Hospitals) were consulted. When an oral formulation could not safely be given via nasogastric or orogastric tube (crushed tablets not recommended by the manufacturer, no other form available), an alternative treatment (drug from the same therapeutic class) was proposed when available.

***Patient-level clinical eligibility* *for an enteral switch* was considered the responsibility of the treating clinician**, who should assess :

1) Is the drug still indicated? Before any change of route is considered the daily medication review must establish whether the medication remains indicated at all and whether it can simply be stopped. De-prescribing takes precedence over route de-escalation: a drug that is no longer needed should be discontinued rather than switched.

2) Organ dysfunction and haemodynamic stability: enteral substitution was considered inappropriate in shock, in patients receiving escalating doses of vasopressors, in the presence of clinical or biochemical signs of splanchnic hypoperfusion, and during the acute resuscitation phase of critical illness, because splanchnic hypoperfusion renders enteral absorption unpredictable. In addition, there are drug-specific or class-specific emergency situations in which the intravenous route must be retained (these appear drug-by-drug in the “Recommendation” column of Table S2). Renal and hepatic dysfunction were considered where they alter the dose rather than the route, and do not in themselves contraindicate substitution.

3) Gastrointestinal function and expected absorption: substitution requires a functioning gastrointestinal tract and a patent, correctly positioned enteral access route. The panel required the absence of vomiting, of ileus or clinically significant gastroparesis, of a persistently high gastric residual volume, of bowel obstruction, of active gastrointestinal bleeding, of clinically significant malabsorption or short bowel, and of a high-output proximal enterocutaneous fistula or recent upper gastrointestinal surgery precluding tube use. Where enteral nutrition is already established and tolerated, the panel regarded this as the most reliable single bedside indicator that absorption is likely to be adequate. Prolonged fasting for procedures, planned extubation and unresolved swallowing assessment were considered temporary contraindications.

***Pharmacokinetic, safety and efficacy data:*** For each medication, pharmacokinetic parameters, including oral bioavailability and time to peak concentration (Tmax), were extracted from summaries of product characteristics of originator drugs. When unavailable, data from generic formulations were used. If still missing, a structured PubMed search was performed using the following query: “[drug international nonproprietary name] AND oral bioavailability”, limited to studies in humans and articles in English or French. Data on clinical efficacy and safety, drug-specific information on absorption, administration and monitoring requirements of enteral versus intravenous administration were obtained through a structured literature search of PubMed and major clinical guidelines. Search terms included [drug international nonproprietary name] of each drug combined with “oral”, “enteral”, “intravenous”, “switch”, “pharmacodynamics”, and “ICU”.

***Classification of recommendations:*** a multidisciplinary panel involving four intensivists (CM, ML, MG and AC), three clinical pharmacists (AL, KB, LS) and one nurse (AP) independently assessed the available evidence and reached consensus regarding the appropriateness of IV-to-enteral substitution for a patient considered clinically eligible for enteral administration (cf ***Patient-level clinical eligibility* *for an enteral switch)***. Based on available pharmacokinetic, safety, efficacy, and guideline data, medications were classified into predefined recommendation categories. Medications with adequate and predictable oral bioavailability, a wide therapeutic index, absence of evidence suggesting poorer tolerability of the oral formulation and no requirement for monitoring were classified as Ready for IV-to-enteral substitution. Medications with low or highly variable oral bioavailability, a narrow therapeutic index, a documented risk of therapeutic failure on switching, or any safety concerns, or for which guidelines mandate monitoring, were classified as Requires monitoring before substitution. Each member of the panel assessed the assembled pharmacokinetic, efficacy, safety and guideline data for every medication and assigned a provisional category. Discordant assignments were discussed until consensus was reached. Where consensus could not be reached on the basis of the available evidence, the more cautious category was assigned. No formal Delphi procedure, quantitative scoring system or measure of inter-rater agreement was used, and the process should therefore be described as expert consensus rather than as a validated grading exercise.

This classification was designed as a pragmatic stewardship tool to support bedside decision-making, and should not be interpreted as a formal guideline recommendation or a validated grading system.

***Carbon footprint and cost analysis:*** To estimate the potential environmental and economic impact of IV-to-enteral substitution, we identified the ten most frequently dispensed intravenous medications across participating ICUs in 2024. As a process-based life-cycle assessment for all 54 eligible medications was not feasible within this work, we selected the five highest-volume eligible medications. Among these, five medications fulfilled two criteria: availability of an enteral alternative and a green recommendation for IV-to-enteral substitution (paracetamol, esomeprazole, furosemide, levetiracetam, and amiodarone). For each medication, greenhouse gas emissions, expressed as kilograms of carbon dioxide equivalents (kgCO₂e), were estimated using a process-based life-cycle assessment according to ISO 14040/44 standards and the Carebone® tool developed by *Assistance Publique–Hôpitaux de Paris*. Further details on the Carebone® methodology and application are provided in Supplementary Data**.** The functional unit corresponded to one defined dose administered either intravenously or enterally. The analysis included active pharmaceutical ingredient production, excipients, packaging, transport, waste disposal, and preparation and administration materials (for IV medications: infusion lines, syringes, transfer needles and dilution fluids; for enteral medications: medication crushing bags), with uncertainty intervals. Medication acquisition costs were obtained from hospital procurement data. Annual carbon emissions and costs were calculated using actual medication consumption recorded during 2024.

*Modelling of IV-to-enteral* *substitution scenarios:* Using annual consumption data for the five selected medications, we modelled hypothetical IV-to-enteral substitution scenarios assuming that 25%, 50%, 75%, or 100% of intravenous administrations could be replaced by enteral formulations. For each scenario, projected reductions in greenhouse gas emissions and medication-related costs were calculated as the difference between observed intravenous consumption and the corresponding enteral substitution model. These scenarios were intended to illustrate the potential environmental and economic benefits achievable through progressive implementation of IV-to-enteral stewardship strategies in routine ICU practice. We deliberately restricted the environmental modelling to the five most frequently administered medications meeting predefined eligibility criteria in order to provide a conservative and operational estimate of the benefits achievable through routine IV-to-enteral stewardship.

*Ethics*

According to French regulations, this non-interventional study based on anonymized aggregated medication data did not require formal ethics committee approval.
