# Supplementary material for: Intravenous-to-enteral drug substitution in the ICU: a single-centre analysis of the potential to reduce the environmental footprint of critical care
Source: Crit Care. 2026 Sep 22;30:487. doi: 10.1186/s13054-026-06331-z (PMC13599322; doi:10.1186/s13054-026-06331-z)
Supplement: Supplementary file 3 [file 13054_2026_6331_MOESM2_ESM.docx]

| **Intravenous medication** | **Enteral alternative** |
| --- | --- |
| **Analgesics** | |
| **Acetaminophen** | Effervescent tablet, disintegrating tablet**, s**yrup |
| **Ketoprofen** | *Crushing the tablet is not recommended*  **Alternative:** **ibuprofen:** dispersible capsule**,** granules, syrup |
| **Nefopam** | Enteral administration of the IV formulation** |
| **Tramadol** | Effervescent tablet, disintegrating tablet, oral solution |
| **Morphine hydrochloride** | **Morphine sulphate:** disintegrating tablet, oral solution  **Extended-release morphine:** openable capsule with microgranules |
| **Gastrointestinal medications** | |
| **Pantoprazole** | **Lansoprazole:** Openable capsule, disintegrating tablet  **Esomeprazole:** Disintegrating tablet, openable capsule  **Omeprazole**: Openable capsule |
| **Ondansetron** | Disintegrating tablet, syrup |
| **Metoclopramide** | Oral solution |
| **Phloroglucinol** | Disintegrating tablet |
| **Psychotropic medications** | |
| **Diazepam** | Crushable tablet***,** oral solution |
| **Clonazepam** | Crushable tablet*, oral solution |
| **Clorazepate** | *Opening the capsule is not recommended, no alternative* |
| **Hydroxyzine** | Crushable tablet*, syrup |
| **Haloperidol** | Oral solution |
| **Tiapride** | Oral solution |
| **Chlorpromazine** | Oral solution |
| **Amitriptyline** | Oral solution |
| **Clomipramine** | Crushable tablet* |
| **Antiepileptic medications** | |
| **Levetiracetam** | Oral solution**,** granules**,** crushable tablet* |
| **Lacosamide** | Syrup**,** crushable tablet* |
| **Sodium Valproate** | Syrup, oral solution  **Extended-release valproate:** micro pellets |
| **Phenobarbital** | Crushable tablet |
| **Brivaracetam** | Oral solution |
| **Cardiovascular medications** | |
| **Nicardipine** | *Crushing the tablet is not recommended*  **Alternatives: lercanidipine:** crushable tablet*, **amlodipine:** openable capsule |
| **Urapidil** | **Extended-release** **urapidil**  Openable capsule |
| **Furosemide** | Crushable tablet*, oral solution |
| **Bumetanide** | Crushable tablet |
| **Acetazolamide** | Crushable tablet* |
| **Amiodarone** | Crushable tablet |
| **Acetylsalicylic acid** | Powder for suspension |
| **Labetalol** | Crushable tablet* |
| **Propranolol** | Oral solution, crushable tablet |
| **Atenolol** | Crushable tablet |
| **Corticosteroids et immunosuppressants** | |
| **Methylprednisolone** | **Prednisolone:** disintegrating tablet |
| **Dexamethasone** | Oral solution |
| **Hydrocortisone** | Crushable tablet |
| **Mycophenolate mofetil** | Powder for suspension |
| **Tacrolimus** | Openable capsule, pellets |
| **Antimicrobials** | |
| **Amoxicillin** | Disintegrating tablet, powder for oral suspension |
| **Amoxicillin-clavulanic acid** | Crushable tablet*, powder for oral suspension |
| **Sulfamethoxazole-trimethoprim** | Crushable tablet, oral solution |
| **Ciprofloxacin** | Pellets for oral suspension |
| **Ofloxacin** | *Crushing the tablet is not recommended, no alternative* |
| **Levofloxacin** | Crushable tablet*, oral solution |
| **Linezolid** | Pellets |
| **Spiramycin** | *Crushing the tablet is not recommended, no alternative* |
| **Clindamycin** | Openable capsule* |
| **Doxycycline** | Disintegrating tablet |
| **Rifampicin** | Oral suspension |
| **Ethambutol** | Crushable tablet |
| **Isoniazid** | Oral solution  **Isoniazid/pyrazinamide/rifampicin:** crushable tablet* (fixed-dose combination) |
| **Acyclovir** | Crushable tablet*, oral solution  **Alternative:** **valacyclovir**: *Crushing the tablet is not recommended* |
| **Ganciclovir** | **Alternative:** **valganciclovir**: crushable tablet* |
| **Metronidazole** | Crushable tablet*, oral solution |
| **Isavuconazole** | Openable capsule |
| **Posaconazole** | Oral suspension |
| **Voriconazole** | Powder for oral suspension |
| **Fluconazole** | Powder for oral suspension |
| **Other** | |
| **Dexchlorpheniramine** | *Crushing the tablet is not recommended*  **Alternative:** cetirizine: syrup |
| **Desmopressin** | Disintegrating tablet, intranasal form |
| **Vitamins** | |
| **Thiamine (vitamin B1)** | Crushable tablet* |
| **Pyridoxine (vitamin B6)** | *Crushing the tablet is not recommended, no alternative* |
| **Niacin (vitamin B3)** | **Nicotinamide**: crushable tablet* |
|  | **Alternative: multivitamin complex (Vitamin A+B1+B2+B3+B5+B6+C+D+E+B3):** oral solution |
| **Folates (vitamin B9)** | *Crushing the tablet is not recommended, no alternative* |
| **Electrolytes** | |
| **Potassium chloride** | Openable capsule, enteral administration of the IV formulation  **Potassium gluconate**: syrup |
| **Disodium phosphate** | Oral solution |
| **Magnesium sulphate** | Magnesium pidolate: oral powder, oral solution |
| **Calcium gluconate/ Calcium chloride** | Calcium carbonate: effervescent tablet, pellets, powder for suspension |

**Table S1: Intravenous medications with available oral formulations and their suitability for enteral administration in the intensive care unit**

*This table lists commonly used intravenous (IV) medications in the ICU and their corresponding enteral alternatives via a nasogastric or orogastric tube in the participating institution. Situations in which the intravenous route remains preferred are specified. Recommendations were guided by the national OMéDIT Normandie–SFPC list of crushable and openable medications (last updated December 2025) and a review of the available literature. Where a capsule is described as openable, the intact gastro-resistant granules or pellets may be dispersed in an appropriate vehicle and administered immediately through a tube of adequate calibre; the granules must never be crushed, as this destroys the enteric coating or abolishes the modified-release profile. Tube blockage is a recognised risk and adequate flushing before and after administration is required. Crushing a tablet, opening a capsule, or administering an oral or intravenous formulation through a feeding tube is frequently outside the terms of the marketing authorisation. Such use is unlicensed, must be justified on a case-by-case basis, documented in the prescription, and supported by a validated pharmaceutical reference. Equivalent agents of the same therapeutic class should be substituted according to local availability; the choice of agent, but not the principle of substitution, is institution-specific.*

*** ***Treatments guided by information from the Geneva University Hospitals drug database.***

***** off-label oral use***
