# Supplementary material for: Intravenous-to-enteral drug substitution in the ICU: a single-centre analysis of the potential to reduce the environmental footprint of critical care
Source: Crit Care. 2026 Sep 22;30:487. doi: 10.1186/s13054-026-06331-z (PMC13599322; doi:10.1186/s13054-026-06331-z)
Supplement: Supplementary file 4 [file 13054_2026_6331_MOESM3_ESM.docx]

| **Enteral alternative** | **Oral bioavailability (%)** | **T max (hours)** | **Safety comparing with IV form** | **Efficacy comparing with IV form** | **Recommendation for IV to PO switch** |
| --- | --- | --- | --- | --- | --- |
| **Analgesics** | | | | | |
| Acetaminophen | 63-89 | 0.5-1 | IV form associated with higher incidence of hypotension [1] | Comparable analgesic and antipyretic effects in critically ill patients [2] | Except in severe pain requiring rapid titration |
| Ibuprofen | 80-100 [3] | 1.5 | Comparable | Enhances Recovery After Surgery guidelines recommend the oral rather than IV route for NSAIDs [4] |  |
| Nefopam | 36 [5] | 0.5-1 | Comparable | No evidence of IV superiority |  |
| Tramadol | 70-90 | 2 | Comparable | Comparable analgesic efficacy between oral and intravenous formulations in non-critical populations [6] |  |
| Morphine sulphate | 30 | 2-4 | Comparable | Enteral formulation recommended as soon as the gastrointestinal tract is functional and pain control is stable [7] |  |
| **Gastrointestinal medications** | | | | | |
| Esomeprazole | 77 | 2-2.5 | Comparable, including *C. difficile* infection and ventilator-associated pneumonia [8] | No evidence of IV superiority  for ulcer prophylaxis or prevention of rebleeding after successful endoscopic therapy [9, 10] | Except in high-risk upper gastrointestinal bleeding (Forrest Ia to IIb ulcers) |
| Ondansetron | 60 | 1.5 | Comparable | No evidence of IV superiority.  Similar clinical outcomes between oral and IV in context of postoperative nausea and vomiting [11] | Except in patients with active vomiting |
| Metoclopramide | 80 | 1 [12] | Comparable | No evidence of IV superiority.  Comparable efficacy in context of chemotherapy [13] |  |
| Phloroglucinol | 42 [14] | 0.67-1 | Comparable | Comparable efficacy between oral and parenteral formulations in reducing pain and spasm in non-ICU populations [14] |  |
| **Psychotropic medications** | | | | | |
| Diazepam | 80-100 | 0.5-1.5 | Comparable | Enteral benzodiazepines are effective alternatives for the management of agitation, anxiety, alcohol withdrawal, delirium tremens and seizure prophylaxis [15] [16] | Except in status epilepticus or severe acute agitation |
| Clonazepam | 90 | 1-4 | Comparable |  |  |
| Hydroxyzine | 80 | 2 | Comparable | Enteral hydroxyzine may represent a viable alternative to intravenous sedatives for light sedation [17] |  |
| Haloperidol | 60-70 | 2-6 | IV administration increases the risk of cardiac adverse effects [18] | Psychiatric expert consensus guidelines recommend prioritizing oral formulations over intramuscular routes whenever possible [18] |  |
| Tiapride | 75 | 1 |  |  |  |
| Chlorpromazine | 10-69 [19] | 0.25-0.5 |  |  |  |
| Amitriptyline | 25-50 | 6 | Serious adverse events, including cardiac arrhythmias, have been reported following IV administration [20] | No superiority of the IV route over oral administration has been demonstrated for neuropathic pain, postoperative pain or major depression [20] [21] |  |
| Clomipramine | 35-65 | 4 [22] | Comparable |  |  |
| **Antiepileptic medications** | | | | | |
| Levetiracetam | 100 | 1.3 | Comparable | Comparable efficacy profiles [23] [24] [25] [26] [27]  **Antiepileptic residual monitoring may be particularly valuable** | Except in status epilepticus |
| Lacosamide | 100 | 0.5-4 | Comparable |  |  |
| Sodium valproate | 100 | 1-4 | Comparable |  |  |
| Phenobarbital | 80 | 8 | Comparable |  |  |
| Brivaracetam | 100 | 0.25-3 | Comparable |  |  |
| **Cardiovascular medications** | | | | | |
| Lercanidipine | 100 | 1.5-3 | Comparable | Current guidelines recommend oral route whenever feasible [28] [29].  No superiority of the IV route [30] | Except in hypertensive emergencies [28] [29] |
| Extended release urapidil | 63-80 | 4-6 | Comparable |  |  |
| Furosemide | 65 | 1 | Comparable | No evidence of IV superiority [31].  **Double the oral dose** | Except in acute heart failure [31] |
| Bumetanide | 95 | 0.5 | Comparable | Can be used alternatively to furosemide in current guidelines [32] |  |
| Acetazolamide | 100 [33] | 2-4 [33] | Comparable | Initial correction of diuretic-induced alkalosis may be more rapid with IV form [34] |  |
| Amiodarone | 30-80 | 3-7 | Comparable | No evidence of IV superiority | Except in emergency atrial fibrillation cardioversion with hemodynamic instability [35] |
| Acetylsalicylic acid | <60 | 0,5-0,67 | Comparable | No difference in clinical outcomes in context of acute coronary syndrome [36]. Current guidelines recommend oral route whenever feasible (platelet inhibition obtained more rapidly) [37] |  |
| Labetalol | 25 | 1-2 | Comparable | Current guidelines recommend oral route whenever feasible (including early acute coronary syndrome management to reduce risk of reinfarction and ventricular arrhythmias) [38] |  |
| Propranolol | 25 [39] | 1-4 | Comparable |  |  |
| Atenolol | 50 | 3 [40] | Comparable |  |  |
| **Corticosteroids et immunosuppressants** | | | | | |
| Prednisolone | 82-89 | 1,5-2,3 | Comparable | Oral prednisolone was not inferior to IV prednisolone In COPD exacerbation [41] or in asthma [42] or in inflammatory disease flares [43] | Except High-dose bolus for acute inflammatory conditions |
| Dexamethasone | >80 [44] | 1-2 | Comparable | No evidence of IV superiority | Except in meningitis, Shock |
| Hydrocortisone | 100 [45] | 1 | Comparable | No evidence of IV superiority | Except in Shock, adrenal crisis |
| Mycophenolate mofetil | 94 | 1 [46] | Comparable | Equivalent efficacy, even in the immediate period after transplant [47]  **Therapeutic drug monitoring is necessary** | Except within 24h after solid organ transplantation |
| Tacrolimus | 20-25 | 1-3 | IV form carries a higher risk of nephrotoxicity, neurotoxicity and anaphylactoid reaction [48] | Comparable efficacy profiles  but oral administration is associated with higher variability in tacrolimus concentrations in comparison to IV administration [48]  **Therapeutic drug monitoring is necessary** | Except within 24h after solid organ transplantation |
| **Antimicrobials** | | | | | **For all antimicrobials: except in shock or in the initial phase of a severe infections**^#^ |
| ***Antibiotics*** | | | | | |
| Amoxicillin | 70 | 1-2 | Comparable | Switch is recommended^#^  Comparable efficacy between oral amoxicillin-clavulanate and IV third-generation cephalosporin in non-ICU pneumonia [49]. In ICU patients with infection who improved after 72 hours, switching was associated with similar tolerability and mortality [50]  **Beyond 9 g/day, IV administration should be prioritized** |  |
| Amoxicillin-clavulanic acid | 70 [51] | 0.75-2 |  |  |  |
| Sulfamethoxazole-trimethoprim | 90 [52] | 2-4 | Comparable | Switch is recommended^#^  **Doses should be adjusted according to the expected MIC** [53] |  |
| Ciprofloxacin | 70-80 | 1-2 | Comparable | Switch is recommended^#^  In ICU patients with infection who improved after 72 hours, switching was associated with similar tolerability and mortality [50].  **With Staphylococcus aureus or Pseudomonas aeruginosa, higher dosages than 750 mg should be given** [54] |  |
| Levofloxacin | 100 | 1-2 | Comparable |  |  |
| Linezolid | 100 | 1.5-2.2 | Comparable | Switch is recommended^#^  Comparable microbiological and clinical effectiveness between oral and IV administration [55] |  |
| Clindamycin | 90 | 1 | Comparable | Switch is recommended^#^  Specific clinical outcome data in the ICU setting are lacking. |  |
| Doxycycline | 73-93 [56] | 2-4 | Comparable | Switch is recommended^#^  Comparable efficacy profiles in Neuroborreliosis [57] |  |
| Metronidazole | 100 | 1-3 | Comparable | Switch is recommended^#^ |  |
| ***Antituberculosis drugs*** | | | | | |
| Rifampicin | 100 | 2-3 | Comparable | Switch is recommended^#^  Enteral nutrition and ICU conditions may reduce the drug’s absorption [58] [59].  **Therapeutic drug monitoring is necessary** | Except during the first 2 weeks of neuro-meningeal tuberculosis |
| Isoniazid | 100 [60] | 1-2 | Comparable |  |  |
| Ethambutol | 75-80 | 2-3 | Comparable |  |  |
| ***Antiviral therapy*** | | | | | |
| Acyclovir | 54 | 0.75-3 | Comparable | French guidelines recommend oral antiviral therapy for primary episodes and recurrent genital or orofacial herpes simplex infections in immunocompetent patients. Specific clinical outcome data in the ICU setting are lacking.  **Therapeutic drug monitoring can be performed** |  |
| Valgancyclovir | 40–60 | 1-2 | Comparable | No evidence of IV superiority |  |
| ***Antifungal therapy*** | | | | | |
| Fluconazole | 90 | 0.5-1.5 | Comparable | Switch is recommended in candidiasis infections after 5 days [61] |  |
| Posaconazole | 10-17 (fasted conditions)  49-59 (fed conditions) [62] | 4-5 | Comparable | Possible subtherapeutic systemic concentrations in ICU patient with oral form [63] [64]  **Therapeutic drug monitoring is necessary** |  |
| Voriconazole | 96 | 1-2 | Potential accumulation and toxicity of sulfobutylether-β-cyclodextrin with IV formulation in patient with kidney failure. | Enteral nutrition may reduce the drug’s absorption. Variability in antifungal target attainment among critically ill patients [65] [66]  **Therapeutic drug monitoring is necessary** |  |
| Isavuconazole | 98 | 2-3 | Comparable | In critically ill patients, interindividual pharmacokinetic variability and suboptimal drug exposure have been observed. [67] [68]  **Therapeutic drug monitoring is necessary** **and higher enteral dosing regimens should be considered at treatment initiation** |  |
| **Other** | | | | | |
| Cetirizine | 93 | 0.9 | Comparable | Comparable efficacy profiles [69] [70] |  |
| Desmopressin | Intranasal: 10  Oral: 0.25 [71] | Intranasal:1Oral: 0.5-2 | Higher Risk of hyponatremia with IV forms [71] [72] | Similar efficacy between forms [71] [73].  **Use different dosage between form (30-time higher in oral form)** | Except in central diabetes insipidus in head trauma or surgery [74] |
| **Vitamins and electrolytes** | | | | | |
| Thiamine | 70-90 [75] | 1-5 [76] | Comparable | Comparable efficacy profiles [75] |  |
| Nicotinamide (vitamin B3/PP) | >90 [78] | 1-2 [78] | Comparable [79] | Comparable efficacy profiles [79] |  |
| Potassium | 85-95 [80] | 1 [81] | Comparable [82] | Comparable efficacy profiles [82] | Without severe, symptomatic, or life-threatening deficiency requiring rapid correction |
| Phosphate | 60-90 [83] | 1-3 | Comparable [83–85] | Comparable efficacy profiles [83–85] |  |
| Magnesium | 24-76 | NR | Comparable [84] | Comparable efficacy profiles [84] |  |
| Calcium | 20-40 [86] | 2 [87] | Comparable | No evidence of IV superiority |  |

**Table S2. Pharmacokinetic characteristics, comparative safety and efficacy, and recommendations for intravenous-to-enteral switching with bibliographic references**

*Safety and efficacy comparing with IV form:* data summarised in this table are derived from published studies, a substantial proportion of which were not conducted in critically ill patients. only data reporting differences in tolerability or safety between intravenous (IV) and enteral/oral formulations are presented. This table should therefore be read as a synthesis of the best available indirect evidence and not as evidence of safety in critical illness. *Recommendations:* Based on available pharmacokinetic, safety, efficacy, and guideline data, medications were classified into two categories: in green : ready for IV-to-enteral substitution, indicating medications that can generally be switched in clinically stable patients with preserved gastrointestinal, in orange : requires monitoring before substitution, indicating medications for which enteral administration is feasible but requires therapeutic drug monitoring and/or close clinical assessment because of variable pharmacokinetics (orange in the table) .*Abbreviations:* NA: not available, IV: intravenous*; Tmax*: time to peak concentration.

^#^ The French Infectious Disease Society, in collaboration with the French Clinical Pharmacy Society and the Infection Prevention and Antibiotic Resistance Network, has recently proposed a structured algorithm to guide the switch to oral antibiotic therapy: **https://documentation-forum.preventioninfection.fr/Record.htm?Record=19135054124919532369&confirm=on**

**References**

1.	Cantais A, Schnell D, Vincent F, et al (2016) Acetaminophen-Induced Changes in Systemic Blood Pressure in Critically Ill Patients: Results of a Multicenter Cohort Study. Crit Care Med 44:2192–2198. https://doi.org/10.1097/CCM.0000000000001954

2.	Ibrahim T, Gebril A, Nasr MK, et al (2023) Unlocking the Optimal Analgesic Potential: A Systematic Review and Meta-Analysis Comparing Intravenous, Oral, and Rectal Paracetamol in Equivalent Doses. Cureus 15:e41876. https://doi.org/10.7759/cureus.41876

3.	Bramlage P, Goldis A (2008) Bioequivalence study of three ibuprofen formulations after single dose administration in healthy volunteers. BMC Pharmacol 8:18. https://doi.org/10.1186/1471-2210-8-18

4.	Beverly A, Kaye AD, Ljungqvist O, Urman RD (2017) Essential Elements of Multimodal Analgesia in Enhanced Recovery After Surgery (ERAS) Guidelines. Anesthesiol Clin 35:e115–e143. https://doi.org/10.1016/j.anclin.2017.01.018

5.	Aymard G, Warot D, Démolis P, et al (2003) Comparative pharmacokinetics and pharmacodynamics of intravenous and oral nefopam in healthy volunteers. Pharmacol Toxicol 92:279–286. https://doi.org/10.1034/j.1600-0773.2003.920605.x

6.	Grond S, Sablotzki A (2004) Clinical pharmacology of tramadol. Clin Pharmacokinet 43:879–923. https://doi.org/10.2165/00003088-200443130-00004

7.	Devlin JW, Skrobik Y, Gélinas C, et al (2018) Clinical Practice Guidelines for the Prevention and Management of Pain, Agitation/Sedation, Delirium, Immobility, and Sleep Disruption in Adult Patients in the ICU. Critical Care Medicine 46:e825. https://doi.org/10.1097/CCM.0000000000003299

8.	Lee P, Fike D, Yang H, et al (2021) Do the types and routes of proton pump inhibitor treatments affect clostridium difficile in ICU patients? A retrospective cohort study. Expert Rev Clin Pharmacol 14:399–404. https://doi.org/10.1080/17512433.2021.1890582

9.	Gralnek IM, Stanley AJ, Morris AJ, et al (2021) Endoscopic diagnosis and management of nonvariceal upper gastrointestinal hemorrhage (NVUGIH): European Society of Gastrointestinal Endoscopy (ESGE) Guideline - Update 2021. Endoscopy 53:300–332. https://doi.org/10.1055/a-1369-5274

10.	Tsai J-J, Hsu Y-C, Perng C-L, Lin H-J (2009) Oral or intravenous proton pump inhibitor in patients with peptic ulcer bleeding after successful endoscopic epinephrine injection. Br J Clin Pharmacol 67:326–332. https://doi.org/10.1111/j.1365-2125.2008.03359.x

11.	Grover VK, Mathew PJ, Hegde H (2009) Efficacy of orally disintegrating ondansetron in preventing postoperative nausea and vomiting after laparoscopic cholecystectomy: a randomised, double-blind placebo controlled study. Anaesthesia 64:595–600. https://doi.org/10.1111/j.1365-2044.2008.05860.x

12.	Graffner C, Lagerström PO, Lundborg P, Rönn O (1979) Pharmacokinetics of metoclopramide intravenously and orally determined by liquid chromatography. Br J Clin Pharmacol 8:469–474. https://doi.org/10.1111/j.1365-2125.1979.tb01028.x

13.	Anthony LB, Krozely MG, Woodward NJ, et al (1986) Antiemetic effect of oral versus intravenous metoclopramide in patients receiving cisplatin: a randomized, double-blind trial. J Clin Oncol 4:98–103. https://doi.org/10.1200/JCO.1986.4.1.98

14.	Corvino A, Magli E, Minale M, et al (2023) Phloroglucinol-Derived Medications are Effective in Reducing Pain and Spasms of Urinary and Biliary Tracts: Results of Phase 3 Multicentre, Open-Label, Randomized, Comparative Studies of Clinical Effectiveness and Safety. Adv Ther 40:619–640. https://doi.org/10.1007/s12325-022-02347-3

15.	Rey E, Tréluyer JM, Pons G (1999) Pharmacokinetic optimization of benzodiazepine therapy for acute seizures. Focus on delivery routes. Clin Pharmacokinet 36:409–424. https://doi.org/10.2165/00003088-199936060-00003

16.	Dhillon S, Oxley J, Richens A (1982) Bioavailability of diazepam after intravenous, oral and rectal administration in adult epileptic patients. Br J Clin Pharmacol 13:427–432. https://doi.org/10.1111/j.1365-2125.1982.tb01397.x

17.	Mistraletti G, Umbrello M, Salini S, et al (2019) Enteral versus intravenous approach for the sedation of critically ill patients: a randomized and controlled trial. Crit Care 23:3. https://doi.org/10.1186/s13054-018-2280-x

18.	Garriga M, Pacchiarotti I, Kasper S, et al (2016) Assessment and management of agitation in psychiatry: Expert consensus. The World Journal of Biological Psychiatry 17:86–128. https://doi.org/10.3109/15622975.2015.1132007

19.	Dahl SG, Strandjord RE (1977) Pharmacokinetics of chlorpromazine after single and chronic dosage. Clin Pharmacol Ther 21:437–448. https://doi.org/10.1002/cpt1977214437

20.	Deisenhammer EA, Whitworth AB, Geretsegger C, et al (2000) Intravenous versus oral administration of amitriptyline in patients with major depression. J Clin Psychopharmacol 20:417–422. https://doi.org/10.1097/00004714-200008000-00005

21.	Faravelli C, Broadhurst AD, Ambonetti A, et al (1983) Double-blind trial with oral versus intravenous clomipramine in primary depression. Biol Psychiatry 18:695–706

22.	Müller FO, Schall R, Mogilnicka EM, et al (1996) Relative bioavailability of four clomipramine hydrochloride tablet products. Biopharm Drug Dispos 17:81–90. https://doi.org/10.1002/(SICI)1099-081X(199601)17:1%3C81::AID-BDD939%3E3.0.CO;2-5

23.	Toublanc N, Du X, Liu Y, et al (2015) Pharmacokinetics, Safety and Bioequivalence of Levetiracetam Intravenous Infusion and Oral Tablets in Healthy Chinese Subjects. Clin Drug Investig 35:495–503. https://doi.org/10.1007/s40261-015-0303-9

24.	Biton V, Rosenfeld WE, Whitesides J, et al (2008) Intravenous lacosamide as replacement for oral lacosamide in patients with partial-onset seizures. Epilepsia 49:418–424. https://doi.org/10.1111/j.1528-1167.2007.01317.x

25.	Limdi NA, Knowlton RK, Cofield SS, et al (2007) Safety of rapid intravenous loading of valproate. Epilepsia 48:478–483. https://doi.org/10.1111/j.1528-1167.2007.00989.x

26.	Kikuchi K, Hamano S-I, Oritsu T, et al (2011) Effectiveness and safety of non-intravenous high-dose phenobarbital therapy for intractable epilepsy during childhood. Brain Dev 33:379–383. https://doi.org/10.1016/j.braindev.2010.07.009

27.	Martellino C, Salafica G, Tripepi G, et al (2025) The efficacy of switching intravenous to oral brivaracetam following status epilepticus: A real-world, multicenter Sicilian study. Epilepsy Behav 172:110744. https://doi.org/10.1016/j.yebeh.2025.110744

28.	Mancia G, Fagard R, Narkiewicz K, et al (2013) 2013 ESH/ESC guidelines for the management of arterial hypertension: the Task Force for the Management of Arterial Hypertension of the European Society of Hypertension (ESH) and of the European Society of Cardiology (ESC). Eur Heart J 34:2159–2219. https://doi.org/10.1093/eurheartj/eht151

29.	Whelton PK, Carey RM, Aronow WS, et al (2018) 2017 ACC/AHA/AAPA/ABC/ACPM/AGS/APhA/ASH/ASPC/NMA/PCNA Guideline for the Prevention, Detection, Evaluation, and Management of High Blood Pressure in Adults: Executive Summary: A Report of the American College of Cardiology/American Heart Association Task Force on Clinical Practice Guidelines. Hypertension 71:1269–1324. https://doi.org/10.1161/HYP.0000000000000066

30.	Reddy P, Yeh Y-C (2009) Use of injectable nicardipine for neurovascular indications. Pharmacotherapy 29:398–409. https://doi.org/10.1592/phco.29.4.398

31.	McDonagh TA, Metra M, Adamo M, et al (2021) 2021 ESC Guidelines for the diagnosis and treatment of acute and chronic heart failure. Eur Heart J 42:3599–3726. https://doi.org/10.1093/eurheartj/ehab368

32.	McDonagh TA, Metra M, Adamo M, et al (2021) 2021 ESC Guidelines for the diagnosis and treatment of acute and chronic heart failure. Eur Heart J 42:3599–3726. https://doi.org/10.1093/eurheartj/ehab368

33.	Yakatan GJ, Frome EL, Leonard RG, et al (1978) Bioavailability of acetazolamide tablets. J Pharm Sci 67:252–256. https://doi.org/10.1002/jps.2600670233

34.	Addison JD, Peterson EJ, Meyenburg L (2023) Intravenous or Oral Acetazolamide for Treatment of Diuretic-Induced Alkalosis in Patients With Heart Failure. Ann Pharmacother 57:1241–1247. https://doi.org/10.1177/10600280231154603

35.	Van Gelder IC, Rienstra M, Bunting KV, et al (2024) 2024 ESC Guidelines for the management of atrial fibrillation developed in collaboration with the European Association for Cardio-Thoracic Surgery (EACTS). Eur Heart J 45:3314–3414. https://doi.org/10.1093/eurheartj/ehae176

36.	Kaur H, Sarma P, Bhattacharyya A, et al (2023) Role of intravenous aspirin versus oral aspirin in the treatment of acute coronary syndrome: Answering a clinical query by systematic review and meta-analysis of randomized controlled trials. Indian J Pharmacol 55:133–137. https://doi.org/10.4103/ijp.ijp_1147_20

37.	Byrne RA, Rossello X, Coughlan JJ, et al (2023) 2023 ESC Guidelines for the management of acute coronary syndromes. Eur Heart J 44:3720–3826. https://doi.org/10.1093/eurheartj/ehad191

38.	Rao SV, O’Donoghue ML, Ruel M, et al (2025) 2025 ACC/AHA/ACEP/NAEMSP/SCAI Guideline for the Management of Patients With Acute Coronary Syndromes: A Report of the American College of Cardiology/American Heart Association Joint Committee on Clinical Practice Guidelines. Circulation 151:e771–e862. https://doi.org/10.1161/CIR.0000000000001309

39.	Duchateau GS, Zuidema J, Merkus FW (1986) Bioavailability of propranolol after oral, sublingual, and intranasal administration. Pharm Res 3:108–111. https://doi.org/10.1023/A:1016345504153

40.	Mcainsh J, Simpson WT, Holmes BF, et al (1980) Bioavailability of atenolol formulations. Biopharm Drug Dispos 1:323–332. https://doi.org/10.1002/bdd.2510010604

41.	de Jong YP, Uil SM, Grotjohan HP, et al (2007) Oral or IV Prednisolone in the Treatment of COPD Exacerbations. Chest 132:1741–1747. https://doi.org/10.1378/chest.07-0208

42.	Jónsson S, Kjartansson G, Gíslason D, Helgason H (1988) Comparison of the oral and intravenous routes for treating asthma with methylprednisolone and theophylline. Chest 94:723–726. https://doi.org/10.1378/chest.94.4.723

43.	Le Page E, Veillard D, Laplaud DA, et al (2015) Oral versus intravenous high-dose methylprednisolone for treatment of relapses in patients with multiple sclerosis (COPOUSEP): a randomised, controlled, double-blind, non-inferiority trial. Lancet 386:974–981. https://doi.org/10.1016/S0140-6736(15)61137-0

44.	Bashir Q, Acosta M (2020) Comparative Safety, Bioavailability, and Pharmacokinetics of Oral Dexamethasone, 4-mg and 20-mg Tablets, in Healthy Volunteers Under Fasting and Fed Conditions: A Randomized Open-label, 3-way Crossover Study. Clin Lymphoma Myeloma Leuk 20:768–773. https://doi.org/10.1016/j.clml.2020.06.022

45.	Johnson TN, Whitaker MJ, Keevil B, Ross RJ (2018) Bioavailability of Oral Hydrocortisone Corrected for Binding Proteins and Measured by LC-MS/MS Using Serum Cortisol and Salivary Cortisone. J Bioequivalence Bioavailab 10:001–003. https://doi.org/10.4172/jbb.1000365

46.	Bullingham R, Monroe S, Nicholls A, Hale M (1996) Pharmacokinetics and bioavailability of mycophenolate mofetil in healthy subjects after single-dose oral and intravenous administration. J Clin Pharmacol 36:315–324. https://doi.org/10.1002/j.1552-4604.1996.tb04207.x

47.	Pescovitz MD, Conti D, Dunn J, et al (2000) Intravenous mycophenolate mofetil: safety, tolerability, and pharmacokinetics. Clin Transplant 14:179–188. https://doi.org/10.1034/j.1399-0012.2000.140301.x

48.	van Dommelen JEM, Grootjans H, Uijtendaal EV, et al (2024) Tacrolimus Variability and Clinical Outcomes in the Early Post-lung Transplantation Period: Oral Versus Continuous Intravenous Administration. Clin Pharmacokinet 63:683–693. https://doi.org/10.1007/s40262-024-01368-1

49.	Dinh A, Duran C, Ropers J, et al (2024) Exclusive oral antibiotic treatment for hospitalized community-acquired pneumonia: a post-hoc analysis of a randomized clinical trial. Clin Microbiol Infect 30:1020–1028. https://doi.org/10.1016/j.cmi.2024.05.003

50.	Oliveira D dos S, da Rocha JV, Gasparetto J, et al (2025) Enteral versus intravenous antibiotics for critically ill patients: A pilot study. The Brazilian Journal of Infectious Diseases 29:104538. https://doi.org/10.1016/j.bjid.2025.104538

51.	Veeraraghavan B, Bakthavatchalam YD, Sahni RD (2021) Orally Administered Amoxicillin/Clavulanate: Current Role in Outpatient Therapy. Infect Dis Ther 10:15–25. https://doi.org/10.1007/s40121-020-00374-7

52.	Chin TW, Vandenbroucke A, Fong IW (1995) Pharmacokinetics of trimethoprim-sulfamethoxazole in critically ill and non-critically ill AIDS patients. Antimicrob Agents Chemother 39:28–33. https://doi.org/10.1128/AAC.39.1.28

53.	Brown GR (2014) Cotrimoxazole - optimal dosing in the critically ill. Ann Intensive Care 4:13. https://doi.org/10.1186/2110-5820-4-13

54.	Debon R, Breilh D, Boselli E, et al (2002) Pharmacokinetic parameters of ciprofloxacin (500 mg/5 mL) oral suspension in critically ill patients with severe bacterial pneumonia: a comparison of two dosages. J Chemother 14:175–180. https://doi.org/10.1179/joc.2002.14.2.175

55.	Liu Y, Liu T, Xiao K, et al (2024) Effects of Oral Versus Intravenous Linezolid Administration on Treatment Effect and Incidence of Thrombocytopenia in Patients with Severe Infection. Intensive Care Res 4:171–179. https://doi.org/10.1007/s44231-024-00071-w

56.	Hopkins AM, Wojciechowski J, Abuhelwa AY, et al (2017) Population Pharmacokinetic Model of Doxycycline Plasma Concentrations Using Pooled Study Data. Antimicrob Agents Chemother 61:e02401-16. https://doi.org/10.1128/AAC.02401-16

57.	Kortela E, Kanerva MJ, Puustinen J, et al (2021) Oral Doxycycline Compared to Intravenous Ceftriaxone in the Treatment of Lyme Neuroborreliosis: A Multicenter, Equivalence, Randomized, Open-label Trial. Clin Infect Dis 72:1323–1331. https://doi.org/10.1093/cid/ciaa217

58.	Koegelenberg CFN, Nortje A, Lalla U, et al (2013) The pharmacokinetics of enteral antituberculosis drugs in patients requiring intensive care. S Afr Med J 103:394–398. https://doi.org/10.7196/samj.6344

59.	Beraldi-Magalhaes F, Parker SL, Sanches C, et al (2023) Is the Pharmacokinetics of First-Line Anti-TB Drugs a Cause of High Mortality Rates in TB Patients Admitted to the ICU? A Non-Compartmental Pharmacokinetic Analysis. Trop Med Infect Dis 8:312. https://doi.org/10.3390/tropicalmed8060312

60.	Zwolska Z, Augustynowicz-Kopeć E, Niemirowska-Mikulska H (2002) The pharmacokinetic factors and bioavailability of rifampicin, isoniazid and pyrazinamid fixed in one dose capsule. Acta Pol Pharm 59:448–452

61.	Cornely OA, Sprute R, Bassetti M, et al (2025) Global guideline for the diagnosis and management of candidiasis: an initiative of the ECMM in cooperation with ISHAM and ASM. Lancet Infect Dis 25:e280–e293. https://doi.org/10.1016/S1473-3099(24)00749-7

62.	Chen L, Krekels EHJ, Heijnen AR, et al (2023) An Integrated Population Pharmacokinetic Analysis for Posaconazole Oral Suspension, Delayed-Release Tablet, and Intravenous Infusion in Healthy Volunteers. Drugs 83:75–86. https://doi.org/10.1007/s40265-022-01819-8

63.	Ray J, Campbell L, Rudham S, et al (2011) Posaconazole plasma concentrations in critically ill patients. Ther Drug Monit 33:387–392. https://doi.org/10.1097/FTD.0b013e31821fb197

64.	König C, Göpfert M, Kluge S, Wichmann D (2023) Posaconazole exposure in critically ill ICU patients: a need for action. Infection 51:1767–1772. https://doi.org/10.1007/s15010-023-02078-9

65.	Roberts JA, Sime FB, Lipman J, et al (2025) Are contemporary antifungal doses sufficient for critically ill patients? Outcomes from an international, multicenter pharmacokinetics study for Screening Antifungal Exposure in Intensive Care Units-the SAFE-ICU study. Intensive Care Med 51:302–317. https://doi.org/10.1007/s00134-025-07793-5

66.	Coste A, Aubron C, Ansart S, et al (2025) Factors affecting voriconazole pharmacokinetic variability in critically ill patients: a systematic review. Crit Care 29:536. https://doi.org/10.1186/s13054-025-05697-w

67.	Jansen AME, Mertens B, Spriet I, et al (2023) Population Pharmacokinetics of Total and Unbound Isavuconazole in Critically Ill Patients: Implications for Adaptive Dosing Strategies. Clin Pharmacokinet 62:1701–1711. https://doi.org/10.1007/s40262-023-01305-8

68.	Rabault C, Bailly S, Thy M, et al (2025) Suboptimal concentrations of isavuconazole in critically ill patients treated for mold infections: a population pharmacokinetic analysis. Expert Opin Drug Metab Toxicol 21:1125–1130. https://doi.org/10.1080/17425255.2025.2545920

69.	Coimbra J, Puntes M, Molina P, et al (2024) Comparative inhibition by oral bilastine, parenteral dexchlorpheniramine, and a new bilastine parenteral (i.v. and i.m.) formulation of histamine-induced wheal and flare response: A randomised phase I trial. Eur J Pharm Sci 203:106900. https://doi.org/10.1016/j.ejps.2024.106900

70.	Pechadre JC, Beudin P, Eschalier A, et al (1991) A comparison of central and peripheral effects of cetirizine and loratadine. J Int Med Res 19:289–295. https://doi.org/10.1177/030006059101900401

71.	LAM KSL, WAT MS, CHOI KL, et al (1996) Pharmacokinetics, pharmacodynamics, long-term efficacy and safety of oral 1-deamino-8-d-arginine vasopressin in adult patients with central diabetes insipidus. Br J Clin Pharmacol 42:379–385. https://doi.org/10.1046/j.1365-2125.1996.39914.x

72.	Zheng L, Cai Z, Shao L, et al (2025) Route-specific effects of desmopressin on bleeding and hyponatremia after kidney biopsy: meta-analysis of intranasal vs. intravenous administration. Front Nephrol 5:1645418. https://doi.org/10.3389/fneph.2025.1645418

73.	McCormick MC, Siripong N, Cooper JD (2018) Desmopressin stimulation testing: Response to intravenous and intranasal forms. Haemophilia 24:e194–e198. https://doi.org/10.1111/hae.13452

74.	Maggiore U, Picetti E, Antonucci E, et al (2009) The relation between the incidence of hypernatremia and mortality in patients with severe traumatic brain injury. Crit Care 13:R110. https://doi.org/10.1186/cc7953

75.	Manzanares W, Hardy G (2011) Thiamine supplementation in the critically ill. Curr Opin Clin Nutr Metab Care 14:610–617. https://doi.org/10.1097/MCO.0b013e32834b8911

76.	Greb A, Bitsch R (1998) Comparative bioavailability of various thiamine derivatives after oral administration. Int J Clin Pharmacol Ther 36:216–221

77.	Stach K, Stach W, Augoff K (2021) Vitamin B6 in Health and Disease. Nutrients 13:3229. https://doi.org/10.3390/nu13093229

78.	Sugandhi VV, Pangeni R, Vora LK, et al (2023) Pharmacokinetics of vitamin dosage forms: A complete overview. Food Sci Nutr 12:48–83. https://doi.org/10.1002/fsn3.3787

79.	Berger MM, Shenkin A, Dizdar OS, et al (2024) ESPEN practical short micronutrient guideline. Clinical Nutrition 43:825–857. https://doi.org/10.1016/j.clnu.2024.01.030

80.	Palmer BF (2015) Regulation of Potassium Homeostasis. Clin J Am Soc Nephrol 10:1050–1060. https://doi.org/10.2215/CJN.08580813

81.	Li X, Qu M, Li H, Li T (2025) Plasma pharmacokinetics of the combination of potassium chloride extended-release tablets and potassium citrate granules in patients with cardiovascular emergencies. Front Cardiovasc Med 12:1636090. https://doi.org/10.3389/fcvm.2025.1636090

82.	DeCarolis DD, Kim GM, Rector TS, Ishani A (2016) Comparative dose response using the intravenous versus enteral route of administration for potassium replenishment. Intensive Crit Care Nurs 36:17–23. https://doi.org/10.1016/j.iccn.2015.08.005

83.	Geerse DA, Bindels AJ, Kuiper MA, et al (2010) Treatment of hypophosphatemia in the intensive care unit: a review. Crit Care 14:R147. https://doi.org/10.1186/cc9215

84.	Kraft MD, Btaiche IF, Sacks GS, Kudsk KA (2005) Treatment of electrolyte disorders in adult patients in the intensive care unit. Am J Health Syst Pharm 62:1663–1682. https://doi.org/10.2146/ajhp040300

85.	Nguyen CD, Panganiban HP, Fazio T, et al (2024) A Randomized Noninferiority Trial to Compare Enteral to Parenteral Phosphate Replacement on Biochemistry, Waste, and Environmental Impact and Healthcare Cost in Critically Ill Patients With Mild to Moderate Hypophosphatemia. Crit Care Med 52:1054–1064. https://doi.org/10.1097/CCM.0000000000006255

86.	Bushinsky DA, Monk RD (1998) Electrolyte quintet: Calcium. Lancet 352:306–311. https://doi.org/10.1016/s0140-6736(97)12331-5

87.	Wang H, Bua P, Capodice J (2014) A comparative study of calcium absorption following a single serving administration of calcium carbonate powder versus calcium citrate tablets in healthy premenopausal women. Food Nutr Res 58:. https://doi.org/10.3402/fnr.v58.23229
