# Supplementary material for: Intravenous-to-enteral drug substitution in the ICU: a single-centre analysis of the potential to reduce the environmental footprint of critical care
Source: Crit Care. 2026 Sep 22;30:487. doi: 10.1186/s13054-026-06331-z (PMC13599322; doi:10.1186/s13054-026-06331-z)
Supplement: Supplementary file 5 [file 13054_2026_6331_MOESM4_ESM.docx]

|  |  | **Intravenous administration** | | | | | |  | | **Enteral administration** | | | | | |  | |  | |
| --- | --- | --- | --- | --- | --- | --- | --- | --- | --- | --- | --- | --- | --- | --- | --- | --- | --- | --- | --- |
| **Annual number of doses** |  |  | Carbon footprint of drug (kgCO_2_e) | Carbon footprint of AD (kgCO_2_e) | Total carbon footprint per dose (kgCO_2_e) | Annual carbon footprint (kgCO_2_e) |  | |  | | Carbon footprint of drug (kgCO_2_e) | Carbon footprint of AD (kgCO_2_e) | Total carbon footprint per dose (kgCO_2_e) | Annual carbon footprint (kgCO_2_e) |  | | **IV-to-enteral carbon footprint ratio** | |  |
| 20 570 |  | **Paracetamol 1000mg** | 0.390 | 0.242 | 0.632 | 12 998 |  | | **Paracetamol 500mg x2** | | 0.084 | - | 0.084 | 1 728 |  | | 7.5 | |  |
| 7 588 |  | **Amiodarone 150mg x2** | 0.614 | 0.450 | 1.064 | 8 072 |  | | **Amiodarone 200mg x3** | | 0.987 | 0.006 | 0.994 | 7 539 |  | | 1.1 | |  |
| 14 091 |  | **Pantoprazole 40mg** | 0.170 | 0.450 | 0.620 | 8 734 |  | | **Lansoprazole 30mg** | | 0.075 | - | 0.075 | 1 057 |  | | 8.3 | |  |
| 20 797 |  | **Furosemide 20mg** | 0.050 | 0.135 | 0.185 | 3 844 |  | | **Furosemide 40mg** | | 0.069 | 0.006 | 0.075 | 1 560 |  | | 2.5 | |  |
| 5 733 |  | **Levetiracetam 500mg** | 0.250 | 0.450 | 0.700 | 4 012 |  | | **Levetiracetam 500mg** | | 0.056 | 0.006 | 0.062 | 357 |  | | 11.2 | |  |
| *AD: administration devices; IV: intravenous; kgCO_2_e: kilograms of carbon dioxide equivalent.* | | | | | | | | | | | | | | | | | | | |

|  |  | **Intravenous administration** | | | | | |  | | **Enteral administration** | | | | | |  | |  | |
| --- | --- | --- | --- | --- | --- | --- | --- | --- | --- | --- | --- | --- | --- | --- | --- | --- | --- | --- | --- |
| **Annual number of doses** |  |  | Cost of drug (€) | Cost of AD (€) | Total cost per dose (€) | Annual cost (€) |  | |  | | Cost of drug (€) | Cost of AD (€) | Total cost per dose (€) | Annual cost (€) |  | | **IV-to-enteral cost ratio** | |  |
| 20 570 |  | **Paracetamol 1000mg** | 0.690 | 0.360 | 1.050 | 21 599 |  | | **Paracetamol 500mg x2** | | 0.024 | - | 0.024 | 494 |  | | 43.8 | |  |
| 7 588 |  | **Amiodarone 150mg x2** | 10.000 | 1.086 | 11.086 | 84 121 |  | | **Amiodarone 200mg x3** | | 0.192 | 0.030 | 0.222 | 1 685 |  | | 49.9 | |  |
| 14 091 |  | **Pantoprazole 40mg** | 0.610 | 1.086 | 1.696 | 23 898 |  | | **Lansoprazole 30mg** | | 0.070 | - | 0.070 | 986 |  | | 24.2 | |  |
| 20 797 |  | **Furosemide 20mg** | 0.270 | 0.361 | 0.631 | 13 123 |  | | **Furosemide 40mg** | | 0.021 | 0.030 | 0.051 | 1 061 |  | | 12.4 | |  |
| 5 733 |  | **Levetiracetam 500mg** | 0.565 | 1.086 | 1.651 | 9 465 |  | | **Levetiracetam 500mg** | | 0.042 | 0.030 | 0.072 | 413 |  | | 22.9 | |  |
| *AD: administration devices; IV: intravenous; €: euro.* | | | | | | | | | | | | | | | | | | | |

**Table S3:** Comparison of Carbon footprints and Cost of top five ICU medications: according intravenous or enteral administration route
